# A bibliometric analysis of studies on environmental enrichment spanning 1967–2024: patterns and trends over the years

**DOI:** 10.3389/fnbeh.2024.1501377

**Published:** 2024-12-04

**Authors:** Gaurav Singhal, Bernhard T. Baune

**Affiliations:** ^1^Division of Otolaryngology - Head & Neck Surgery, Department of Surgery, University of Wisconsin, Madison, WI, United States; ^2^Department of Mental Health, University of Münster, Münster, Germany

**Keywords:** environmental enrichment, brain, rodents, hippocampus, learning, memory, behavior, cognition

## Abstract

Environmental Enrichment (EE) has received considerable attention for its potential to enhance cognitive and neurobiological outcomes in animal models. This bibliometric analysis offers a comprehensive evaluation of the EE research spanning from 1967 to 2024, utilizing data extracted from Scopus and analyzed through R and VOSviewer. The volume of publications, citation patterns, and collaborations were systematically reviewed, highlighting important contributions and emerging trends within the field of animal research. Core concepts of EE research are mapped, revealing key themes such as neuroplasticity, cognitive function, and behavioral outcomes. A significant increase in EE research is demonstrated, particularly after the year 2000, reflecting growing scientific and public interest in EE paradigms. This analysis provides insights into the global contributions and collaborative networks that have shaped EE studies over time. The role of EE in advancing the understanding of neurobiological, neurodevelopmental, and neurodegenerative processes is underscored. Influential contributors, leading countries, and high-impact journals in the field of EE are identified, offering a valuable resource for researchers seeking to understand or extend the current knowledge base. The strategic selection of keywords and rigorous data curation methods ensure that the findings accurately reflect the most impactful aspects of EE research in animals. This study serves as an essential reference for future explorations and applications of EE across disciplines. By providing a clear and structured overview of the field, this paper aims to serve as a foundation for ongoing and future research initiatives, encouraging more robust investigations and applications of EE to enhance cognitive and neurological health globally.

## Introduction

The Environmental Enrichment (EE) paradigm has been explored since the 1960s for its role in enhancing neural and cognitive functions in animal models (Vogel et al., 1967). Conventionally, EE includes objects that provide physical, cognitive, sensory, and social stimulation to improve overall mental health (Singhal et al., 2014; Baumans, 2005). EE has been extensively explored for its role in enhancing brain function. Studies have shown that EE increases the size and complexity of dendritic branches (Jung and Herms, 2014; Rojas et al., 2013; Beauquis et al., 2010; Leggio et al., 2005), promotes neurogenesis in the hippocampus (Beauquis et al., 2010; Fabel et al., 2009; Khalil, 2024; Rizzi et al., 2011), restores and upregulates synaptic plasticity (Aghighi Bidgoli et al., 2020; Stein et al., 2016; Kim et al., 2016), improves neurotransmitter function (Mora et al., 2007; Darna et al., 2015; Koh et al., 2007), and enhances expression of neurotrophic factors (Sun et al., 2010; Dandi et al., 2018; Birch et al., 2013). EE has also been shown to help in improving memory (Aghighi Bidgoli et al., 2020; Birch et al., 2013; Sahini et al., 2024), learning (Sahini et al., 2024; Bramati et al., 2023; Zentall, 2021), cognition (Zentall, 2021; Singhal et al., 2019a), and adaptability to stressful environments (Lehmann and Herkenham, 2011; Fan et al., 2021; Cabrera-Álvarez et al., 2024). However, much of the research on EE is somewhat fragmented with studies focusing on different aspects of EE using varied species of animals and methodologies over the years.

Bibliometric analysis is a systematic and quantitative approach to evaluate the current structure, development, and impact of research in specific fields (Ellegaard and Wallin, 2015; Donthu et al., 2021). It offers valuable insights into the trends and patterns of studies. By identifying key research themes, influential studies, and collaborative networks, this analysis helps to integrate diverse research patterns, guide future directions, and foster a more cohesive understanding of research impact and potential applications. To date, no bibliometric analysis has been conducted on EE research in animal models.

Hence, in this study, bibliometric techniques were utilized to analyze past and current research on EE, a growing area of study within neuroscience and related disciplines. By examining the volume of publications, citation patterns, and collaborations among authors and institutions, this analysis maps the scholarly network of EE research, identifies key contributors, and uncovers emerging trends in the field. It highlights the historical progression and scientific impact of EE, offering insights into its influence on fields such as neurodevelopmental disorders, mental health, and animal welfare. This broader perspective is essential for understanding how themes like neuroplasticity, behavior, and stress resilience have developed over time and for recognizing the interdisciplinary applications of EE beyond specific variables like brain function and behavior. By identifying knowledge gaps and underexplored areas, the analysis provides valuable insights to guide future research and advance the field in new directions, making it a crucial resource for researchers across disciplines.

To achieve this, Scopus was used as the database for article retrieval. The metadata of the collected articles were analyzed using R and VOSviewer software, both commonly used in bibliometric analyses (Derviş, 2019; McAllister et al., 2022). This helped us analyze keywords, authorship, co-authorship, co-citation, country distribution, and the impact of scientific journals. The analyses also allowed for the visualization of research networks and thematic clusters, providing valuable insights into the collaborative network and influential works that have shaped the research on EE over time.

## Methodology

Scopus, a scientific abstract and citation database of peer-reviewed literature from the academic publisher Elsevier, was utilized for searching and collecting articles related to EE (Burnham, 2006; Thelwall and Sud, 2022). A bibliometric analysis was then performed using the “Bibliometrix” package in R (version 4.4.1) and VOSviewer (version 1.6.20) to visualize the pooled metadata from the cataloged articles. EndNote (version 20) was used for referencing purposes. Table 1 shows the step-by-step process of data retrieval, handling, processing, analysis, and visualization involved in the bibliometric analysis.

**Table 1:** Steps involved in the bibliometric analysis process: data collection, curation, and visualization.

**#**	**Step**	**Output**	**Tools/software used**
1	Defining the research scope	The focus of the bibliometric analysis was defined, and research questions and objectives were established.	Researcher input
2	Data source selection	An appropriate database for the analysis was chosen, ensuring comprehensive coverage of high-quality literature. Two thousand three hundred and eighty five articles were retrieved on the subject.	Scopus
3	Data curation	The dataset was filtered using specific inclusion/exclusion criteria (e.g., limiting the search to articles, reviews, and English publications). The search was refined with additional keywords. Final dataset with 1,847 articles was retrieved for further analysis.	Manual review complemented the automated search to ensure relevance
4	Keyword consolidation	The synonyms and variations of key terms were merged to reduce redundancy and for ensuring consistent terminology throughout the dataset. Manual judgment was crucial in grouping similar keywords to reflect broader research trends accurately.	Thesaurus file
5	Data export	The relevant metadata from Scopus was exported, including titles, abstracts, keywords, citations, and authors for further analysis.	CSV file containing bibliometric metadata
6	Performance analysis	Key performance metrics such as annual publication counts, corresponding authors and total citations by country were analyzed.	Bibliometrix in R
7	Link strength analysis	Conducted link strength analysis to explore keyword co-occurrence, co-authorship, co-citation, and collaborations across different entities.	VOSviewer
8	Network visualization	Visualized the bibliometric results. Visual outputs highlight dominant clusters and key entities.	VOSviewer
9	Interpretation and reporting	Provided a comprehensive discussion on the findings and emerging trends, integrating insights from link strengths and network visualization.	Subjective assessment

### Database selection

Scopus was selected as the primary database for data collection due to its comprehensive coverage of peer-reviewed, high-quality research publications and its strong reputation within the academic community (Burnham, 2006; Thelwall and Sud, 2022). Scopus provides robust indexing of a wide range of disciplines, including neuroscience, which is central to the topic of EE. It offers advanced citation tracking and a broad selection of journals, ensuring that the analysis captures key publications and the most influential studies in the field.

Google Scholar, while more comprehensive in terms of the range of documents indexed, does not offer the same level of quality control. Similarly, PubMed, while highly respected for biomedical research, is too specialized for the broader scope of EE. Additionally, Web of Science was excluded from the data collection sources due to its overlapping coverage with Scopus. By focusing on a single source, Scopus, efficiency in data handling was maintained while still capturing a comprehensive view of the relevant literature without the redundancy that might arise from using multiple similar databases.

### Data curation

The primary search terms used during the database queries were “environmental enrichment” and related terms, such as “enriched environment,” ensuring that the search remained centered around the core theme of the analysis, enhancing the accuracy of the retrieved dataset. This search yielded a total of 2,385 articles. The time range of the articles spanned from 1967 to August 2024. Unrelated subject areas such as Engineering, Social Sciences, Chemical Engineering, Earth and Planetary Sciences, Chemistry, Computer Science, Physics and Astronomy, Nursing, Materials Science, Energy, Arts and Humanities, Mathematics, Business Management and Accounting, Decision Sciences, and Dentistry were excluded from the Scopus search. The selection was limited to documents categorized as articles, reviews, and book chapters, and source types were restricted to journals, books, and book series. Additionally, only articles published in English were included.

Titles, and where necessary, abstracts, were reviewed to ascertain their direct relevance to the core themes of the bibliometric analysis. Articles that did not explicitly align with the EE theme were excluded. Specifically, articles were excluded if they did not explicitly focus on the core theme of EE, such as neuroplasticity, behavior, cognition, or other related biological and neurological outcomes. Studies that lacked direct application to the biological, cognitive, or behavioral aspects of EE were also excluded. For example, studies that mentioned “enrichment” in contexts unrelated to neuroscience or behavior (e.g., enrichment in environmental engineering) were excluded to maintain the biological focus of the analysis. Furthermore, studies addressing topics such as animal welfare or habitat design, without examining the biological or neurological effects of EE, were excluded, as they did not contribute to the core focus of the bibliometric analysis on the impact of EE on brain function and behavior. Some articles focusing on broader or less directly related topics, such as education or human animal social interactions, which fall under fields like psychology but may not explicitly deal with the core biological aspects of EE (e.g., neuroplasticity or brain function in animal models), were also excluded. Preliminary analysis using VOSviewer and R helped in the identification of additional keywords, which contributed to further refining the search and extraction of more relevant articles (refer to Appendix 1 for a comprehensive list of keywords). This rigorous selection process narrowed the dataset to 1,847 articles on Scopus, ensuring that retained studies were directly related to the analysis objectives. Appendix 2 shows the exported values from Scopus for each inclusion criteria. The metadata of these articles was then exported from Scopus as a CSV file for subsequent evaluation in R and VOSviewer.

The articles were included regardless of the availability of their full texts. This decision is aligned with the nature of bibliometric analysis, which primarily relies on metadata such as titles, abstracts, keywords, authors, and citation details rather than the full text content. Bibliometric tools like VOSviewer extract and analyze keyword frequencies, citation information, and authors details to uncover patterns and trends in the literature, making the full text of articles less crucial for this type of analysis. Furthermore, this inclusive approach helps mitigate potential bias that could arise from excluding significant studies due to access restrictions.

### Keyword consolidation strategies

To streamline the dataset and reduce redundancy, a systematic approach was adopted using a thesaurus file to merge keywords representing the same concept for visualization purpose in VOSviewer. For instance, enriched environment, environmental enrichment, and environment enrichment were unified under “ee,” mice, mouse, rat, and rats were consolidated under the broader term “rodents,” behavior and behavior were considered together under the term “behavior,” and aging and aging were unified as “aging.” Similarly, anxiety and anxiety-like behavior were grouped under the term “anxiety.” This approach ensured comprehensive coverage of similar keywords written differently. Additionally, the variations in keyword forms, including singular and plural forms, as well as terms with or without apostrophes, were unified to avoid fragmentation. For instance, variations like Alzheimer Disease and Alzheimer's Disease were standardized to “Alzheimer's Disease,” and pig and pigs were merged into “pigs.”

Reducing the number of distinct nodes in the network helped minimizing visual clutter, making dominant research themes more discernible, as well as ensuring that fragmented studies were cohesively analyzed, providing a more accurate representation of the research activity in the field.

### Selection criteria in VOSviewer

While conducting bibliometric analysis in VOSviewer, author keywords were chosen over index keywords to provide a more accurate reflection of the topics that researchers themselves consider central to their studies. Author keywords are directly provided by the original authors of the papers and are therefore more likely to represent the primary concepts and research areas the authors aimed to explore. This contrasts with index keywords, which may be assigned by databases based on broader classification systems and may not always reflect the specific focus of the research.

To ensure the robustness of the analysis, a minimum occurrence threshold of five was set for keywords to be included. This criterion was established to maintain a balance between dominant research topics while excluding terms that appear infrequently and may not be representative of trends in the field. Only keywords with a meaningful level of recurrence across multiple studies were considered, enhancing the reliability and interpretability of the co-occurrence network and thematic clustering generated by the software.

A minimum citation count of five was also established for authors and countries involved in the analysis. Setting a citation count threshold helped to filter out less influential works, focusing on those researchers and countries that have not only been prolific in terms of publication but have also made significant scholarly contributions in the field of EE. This helped us understand which researchers and regions are driving advancements and gaining recognition in this area of study.

Similarly, a minimum document threshold of five was established for authors, countries, and journals to be included in the co-authorship analysis. This threshold was implemented to identify and highlight the most active and collaborative authors, countries, and scientific journals in the field of EE. By doing so, the focus remained on contributors who not only have substantial involvement in EE research but also play pivotal roles in the dissemination and development of knowledge within the global scientific community through collaboration and publications in high-impact journals.

For the co-citation analysis, a minimum citation threshold of 200 was set for authors. Co-citation occurs when two or more authors are cited together in other works, indicating that their research is often referenced in conjunction with each other and may share thematic or methodological similarities. This analysis is instrumental in identifying influential authors whose work forms the basis for ongoing research within the field. The co-citation measure highlights the most impactful contributors whose research has been consistently acknowledged by peers. This complements the other bibliometric analyses, offering a well-rounded view of contemporary collaborations.

Finally, to maintain clarity and focus, the top 25 results in each category were selected for co-occurrence visualization. This methodological choice helped avoid visual clutter and ensures that the most significant contributors in the field are highlighted.

### Link strength and network visualization analysis

Link strength is a central metric in bibliometric and network analysis that quantifies the strength of the association between two elements in a co-occurrence network, such as authors, institutions, or keywords. It provides insight into how closely two elements are connected based on the frequency of their co-occurrence within the dataset. In this study, link strength was calculated to assess the relationships between various entities within EE research, allowing for the identification of influential collaborations, research themes, and trends.

Link strength is not limited to a simple binary relationship (i.e., whether or not two entities are connected) but it provides a continuous measure of the degree of association between entities. For example, a higher link strength between two authors suggests frequent collaboration, while a lower link strength suggests occasional collaboration. Similarly, when analyzing institutional linkages, a strong link indicates active collaboration between institutions on shared research projects, while weaker links reflect less frequent or occasional collaboration. In this study, by applying a threshold to the minimum number of occurrences required for inclusion in the network, only relevant and significant links were analyzed, ensuring that the most meaningful connections were highlighted. This filtering helped avoid the inclusion of less frequent or potentially less relevant associations.

To compute link strength, the software VOSviewer was used to generate co-occurrence networks which is a powerful tool for understanding the dynamics of a research field. In these networks, nodes represent entities (such as authors or institutions), and links between nodes represent the number of times those entities appear together within the same publication. The strength of the link is determined by the frequency of these co-occurrences: the more frequently two entities co-occur, the stronger the link between them. This is represented numerically by the link strength value. The link strength is represented visually as lines or edges connecting nodes, with the size of each node reflecting the frequency or magnitude of that entity's contribution to the dataset and thickness of each line corresponding to the magnitude of the link strength. Thicker lines indicate stronger relationships between entities, while thinner lines represent weaker or less frequent connections. Link strength, size of nodes and thickness of lines help in identifying clusters within the network, where groups of entities are more closely connected to each other than to the rest of the network. These clusters often represent specific research communities that are highly interconnected and are color coded in VOSviewer. For example, in collaboration analysis, different colors may represent various collaborative networks across geographic regions or research subfields. This clustering analysis allows for the identification of dominant research trends, leading contributors, and emergent fields of study. The size of a cluster indicates the significance of that group in the network, while the proximity of clusters to each other reveals the degree of interdisciplinary collaboration or overlap between research topics. A dense network, characterized by numerous and strong connections between nodes, suggests a highly collaborative field where entities frequently interact or co-publish. In contrast, a sparse network with fewer or weaker connections may indicate a more fragmented field, where research is conducted in isolated pockets with less interaction between entities. By quantifying and visualizing link strength and nodes, this study has provided a clear map of the collaborative landscape and intellectual structure within EE research.

## Results

### Annual scientific production of research on EE

**Figure 1:**
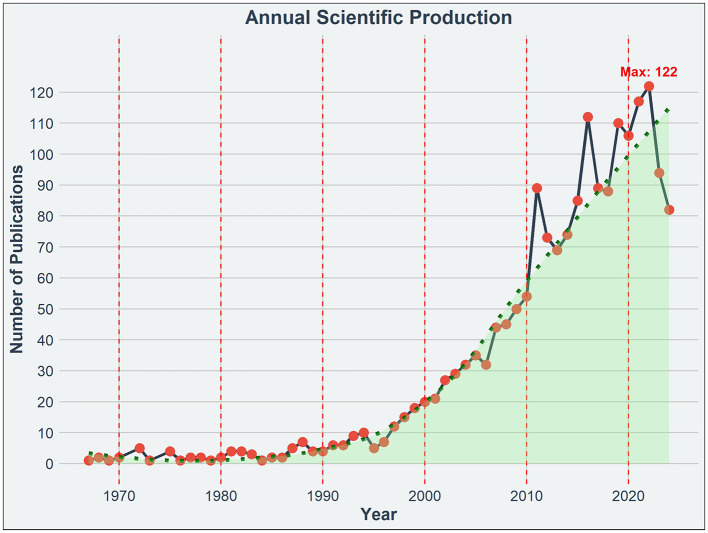
Annual scientific production on EE research. The graph displays the annual number of publications related to EE from 1967 to 2024.

Figure 1 depicts the annual scientific production of research in EE from 1967 through 2024. It shows a modest beginning with fewer than 10 publications annually until the late 1980s, followed by a gradual increase through the 1990s. A notable acceleration in publication volume began in the early 2000s, reflecting growing interest and recognition of EE significance in scientific research. This upward trend continued over the years, reaching a peak of 122 publications in 2022. The trend line from 2000 onwards suggests a period of particularly rapid growth, aligning with heightened scientific and public interest in the implications and applications of EE.

### Corresponding authors by country

**Figure 2:**
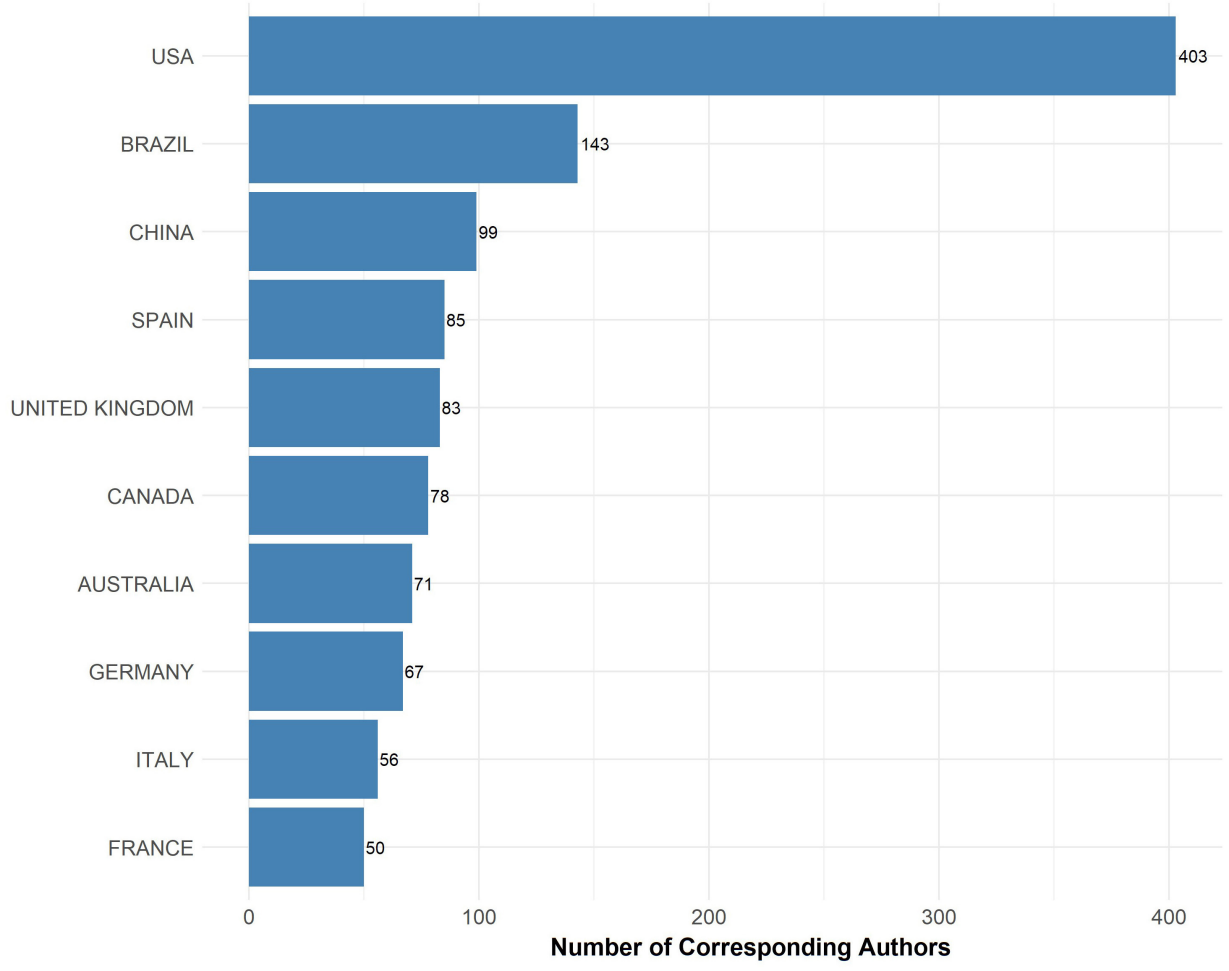
Corresponding author count by country. The bar chart presents the number of corresponding authors contributing to EE research by country.

The bar graph illustrates the distribution of corresponding authors focusing on EE research by country (Figure 2). The USA leads significantly with 403 corresponding authors, demonstrating a dominant role in EE research. Brazil follows with 143 authors, highlighting its substantial contribution to the field, particularly when compared to other nations. China, Spain, and the United Kingdom also show notable involvement with 99, 85, and 83 corresponding authors, respectively, underscoring their active research communities in this area.

Canada, Australia, and Germany contribute with 78, 71, and 67 corresponding authors respectively, reflecting a strong engagement in EE studies. Italy and France, with 56 and 50 corresponding authors respectively, round out the list of the top 10 countries contributing to EE research. This geographic distribution indicates a broad international interest and varied contributions to the advancements in EE, reflecting its global relevance and interdisciplinary nature.

### Total citation by country

**Figure 3:**
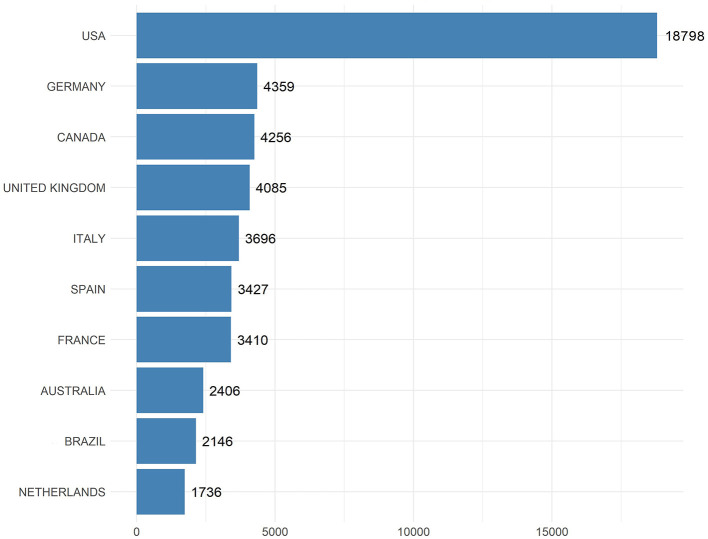
Total citations by country. This bar chart displays the total number of citations received by publications on EE research, categorized by country.

The bar chart illustrates the total number of citations received by all research articles on EE published by authors from various countries (Figure 3). The USA stands out prominently with a staggering 18,798 citations, illustrating its pivotal role and significant impact in EE research globally. Germany follows as a distant second with 4,359 citations, underscoring its substantial contributions to the field.

Canada and the United Kingdom are closely paired with 4,256 and 4,085 citations respectively, indicating their strong academic outputs and influential research in EE. Italy also shows a robust presence in the field with 3,696 citations, followed by Spain and France, which have accumulated 3,427 and 3,410 citations respectively.

Australia, Brazil, and the Netherlands round out the list of top 10 countries with 2,406, 2,146, and 1,736 citations respectively. These figures highlight the broad international interest and the recognition of work on EE originating from these regions by the global scientific community.

### Keywords analysis

A total of 3,538 author keywords were identified, out of which 180 met the threshold criteria of a minimum occurrence of five in VOSviewer. Link strength in this analysis represents the frequency with which two keywords appear together across the dataset. For visualization purposes, the top 25 keywords were selected based on their occurrences and total link strength, indicating how strongly a keyword is associated with others in the network (Table 2).

**Table 2:** Top 25 keywords based on the number of occurrences and total link strength.

**Keyword**	**Occurrences**	**Total link strength**
EE	1,050	1,107
Learning and memory	156	377
Hippocampus	139	315
Behavior	164	292
Rodents	163	272
Anxiety	98	201
Neurogenesis	71	162
tbi	45	152
Morris water maze	38	147
Stress	84	143
Aging	55	120
Cognition	63	119
Functional recovery	27	118
Exercise	52	117
Controlled cortical impact	22	108
Neuroplasticity	62	103
Corticosterone	49	93
Depression	36	93
bdnf	47	91
Alzheimer's disease	47	88
Pigs	52	73
Synaptic plasticity	28	54
Inflammation	16	52
Prefrontal cortex	23	50
Dopamine	28	47

Keywords sorted in order of total link strength from highest to lowest. ee, environmental enrichment; tbi, traumatic brain injury.

The most central keyword, “ee,” appeared 1,050 times and had a total link strength of 1,107, suggesting that “ee” was not only frequently used but also had strong associations with other keywords. Other significant keywords include “behavior” with 164 occurrences and a link strength of 292, and “rodents” with 163 occurrences and a link strength of 272, reflecting the focus on behavioral studies and rodent models in EE research. Keywords such as “learning and memory” and “hippocampus” also featured prominently, with high occurrences and link strengths, underscoring the neurobiological dimensions explored in EE research. Higher link strength for these keywords signifies that they are frequently co-mentioned with other key terms, revealing how interconnected themes like memory and hippocampal function are within the broader scope of EE studies. The selective visualization helped highlight the most influential and recurrent themes within the extensive dataset, providing clear insights into the dominant research areas within the field of EE. By focusing on both occurrences and link strength, the analysis effectively illustrates not only which topics are most frequently studied but also how closely related they are to other research areas, revealing key thematic clusters.

### Keyword network visualization

**Figure 4:**
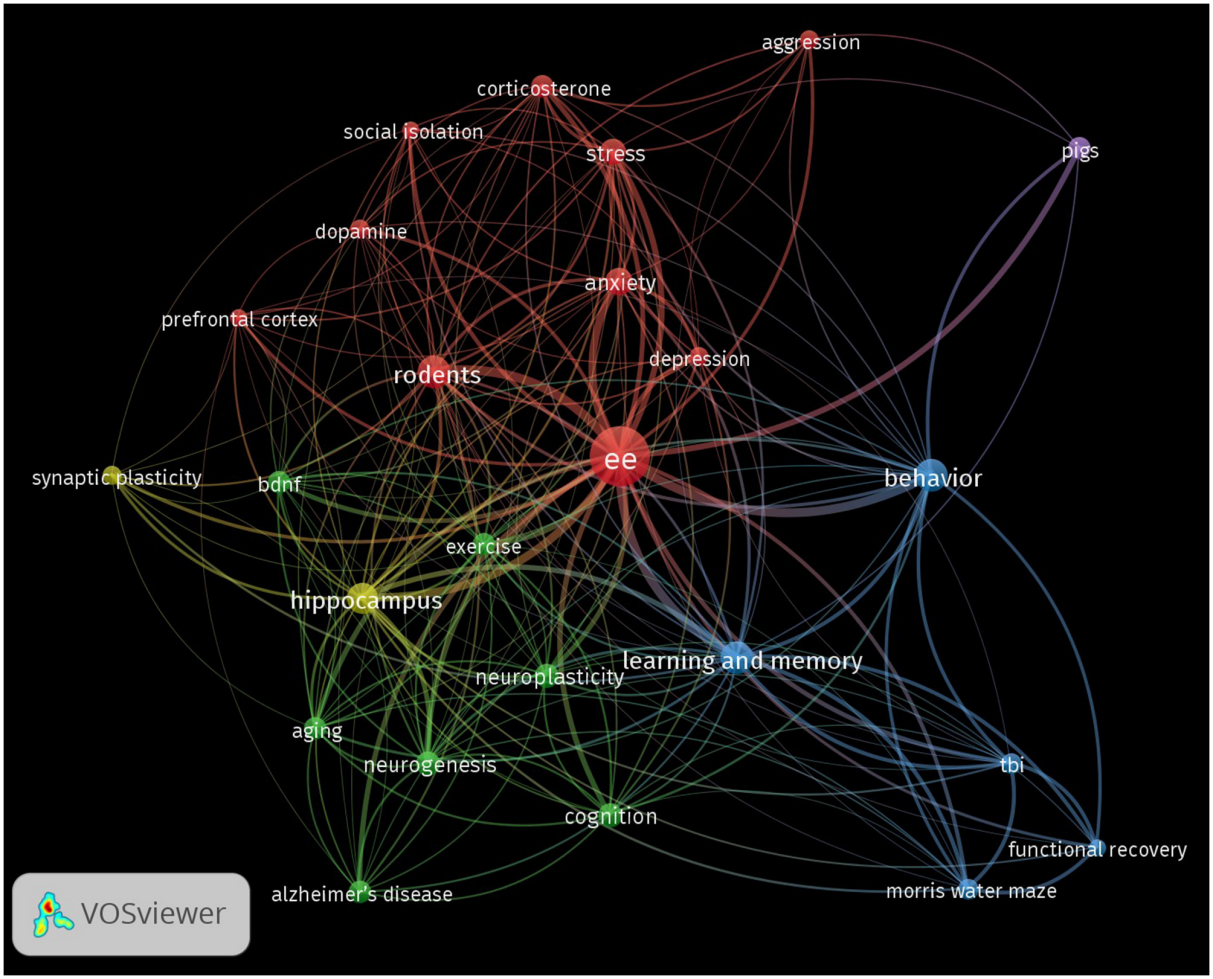
Keyword co-occurrence network in EE research. The network visualization shows the co-occurrence of keywords in EE research articles, with nodes representing individual keywords and edges representing co-occurrences between them. The size of each node corresponds to the frequency of the keyword's appearance, while the color represents different thematic clusters. The thickness of the edges represents the strength of co-occurrence of keywords, with thicker lines indicating a strong and more frequent association.

As expected, the network visualization reveals the term “ee” as the central node. Surrounding this focal point, several key thematic clusters and terms have emerged, highlighting significant areas within the research landscape (Figure 4). In the figure, nodes represent individual keywords, and the size of each node corresponds to the frequency of occurrence of that keyword in the dataset. The lines or edges connecting the nodes represent the co-occurrence of keywords in the same publications. The thickness of these lines corresponds to the strength of association between the terms. Thicker lines indicate that the two keywords frequently appear together, while thinner lines suggest a weaker or less frequent association. The colors of the nodes and links (lines or edges) represent distinct clusters of related terms. The visual clusters formed by the interconnected nodes reveal the primary research domains and highlight the interdisciplinary nature of EE studies across neuroscience, behavior, and recovery from neurodegeneration or injury.

The cluster in green, containing keywords such as “hippocampus,” “synaptic plasticity,” “neuroplasticity,” “bdnf,” and “neurogenesis” are densely connected, emphasizing the significant focus on the neurobiological effects of EE. The strong links between these terms suggest a deep research interest in how EE influences brain structure and function, particularly in areas related to learning, memory, and cognitive resilience. The presence of keywords like “exercise,” “aging,” and “Alzheimer's disease” in this same cluster points to the diverse applications of EE across different life stages and conditions, including gerontology and neurodegenerative diseases.

Similarly, the red cluster captures keywords related to behavioral outcomes, including “behavior,” “anxiety,” “depression,” “stress,” and “aggression.” The connections among these terms illustrate the extensive study of role of EE in modulating behavior and emotional responses, often in the context of stress-related disorders and mental health. The thickness of the edges between these nodes further highlights the strong association and frequent co-mention of these terms in the EE literature.

The blue cluster reflects research focused on the role of EE in recovery from cognitive and physical impairments. Terms like “tbi” (traumatic brain injury), “Morris water maze,” and “functional recovery” are prominent, underscoring the application of EE in cognitive and functional recovery studies, particularly within rodent models.

Interestingly, the term “rodents” appears as a central node connected to both neurobiological and behavioral clusters, indicating the prevalent use of rodent models in EE research. Its connections to experimental outcomes such as the “Morris water maze” reinforce the central role of rodent-based studies in advancing our understanding of the effects of EE on cognition and behavior.

### Author collaboration analysis

During our co-authorship analysis of EE research, a total of 7,339 authors were considered, but only 97 met the threshold of having authored a minimum of five co-authored publications on EE. Link strength in this analysis refers to the strength of the collaborative relationships between authors, measured by the number of publications they co-authored together. The analysis was then visualized focusing on the top 25 authors, chosen based on their number of documents, citations, and the strength of their collaborative links considered together (Table 3).

**Table 3:** Top 25 authors based on the number of documents, citations and total link strength from collaboration work.

**Author**	**Documents**	**Citations**	**Total link strength**
Kline, Anthony E.	31	1,236	65
Hannan, Anthony J.	27	1,613	16
Bardo, Michael T.	20	1,386	3
Cheng, Jeffrey P.	19	604	52
Bondi, Corina O.	19	359	56
Maffei, Lamberto	15	1,678	10
Solinas, Marcello	14	1,162	29
Berardi, Nicoletta	11	1,146	10
Thiriet, Nathalie	11	943	26
Baumans, V.	11	832	25
Jaber, Mohamed	10	1,095	24
Van de Weerd, H.A.	9	790	22
Radabaugh, Hannah L.	9	168	32
Van Loo, P.L.P.	7	578	21
Lardeux, Virginie	7	532	20
Van Zutphen, L.F.M.	6	591	20
Monaco, Christina M.	6	231	22
Koolhaas, J.M.	5	549	18
Folweiler, Kaitlin A.	5	190	18
Leary, Jacob B.	5	161	20
Baune, Bernhard T.	5	149	19
Corrigan, Frances	5	149	19
Jaehne, Emily J.	5	149	19
Singhal, Gaurav	5	149	19
Lajud, Naima	5	115	19

Sorted in order of number of documents on EE in collaboration, from highest to lowest.

Anthony E. Kline emerged as one of the most central figures with 31 documents, reflecting his extensive collaborative network, underscored by the highest total link strength of 65. A higher link strength demonstrates that Kline not only produces a large volume of work but also has deep, established collaborations, making him a pivotal figure in fostering collaborative research within the EE community. Lamberto Maffei also stood out with 15 documents and the highest citation count of 1,678 with co-authors, indicating both prolific output and substantial influence. Although his link strength is not as high as Kline's, his citation count reflects the significance of his research within the collaborative network. Notably, the network also includes other key contributors like Anthony J. Hannan, Marcello Solinas, Jeffery P. Cheng, and Corina O. Bondi, who have been instrumental in expanding the interdisciplinary reach of EE studies. The link strength among these authors signifies frequent and robust collaborations, showing how their joint efforts have driven the field forward. It showcases the deep interconnections between authors that foster scientific inquiry and highlights the importance of collaboration in advancing research within the EE community.

### Author collaboration network visualization

**Figure 5:**
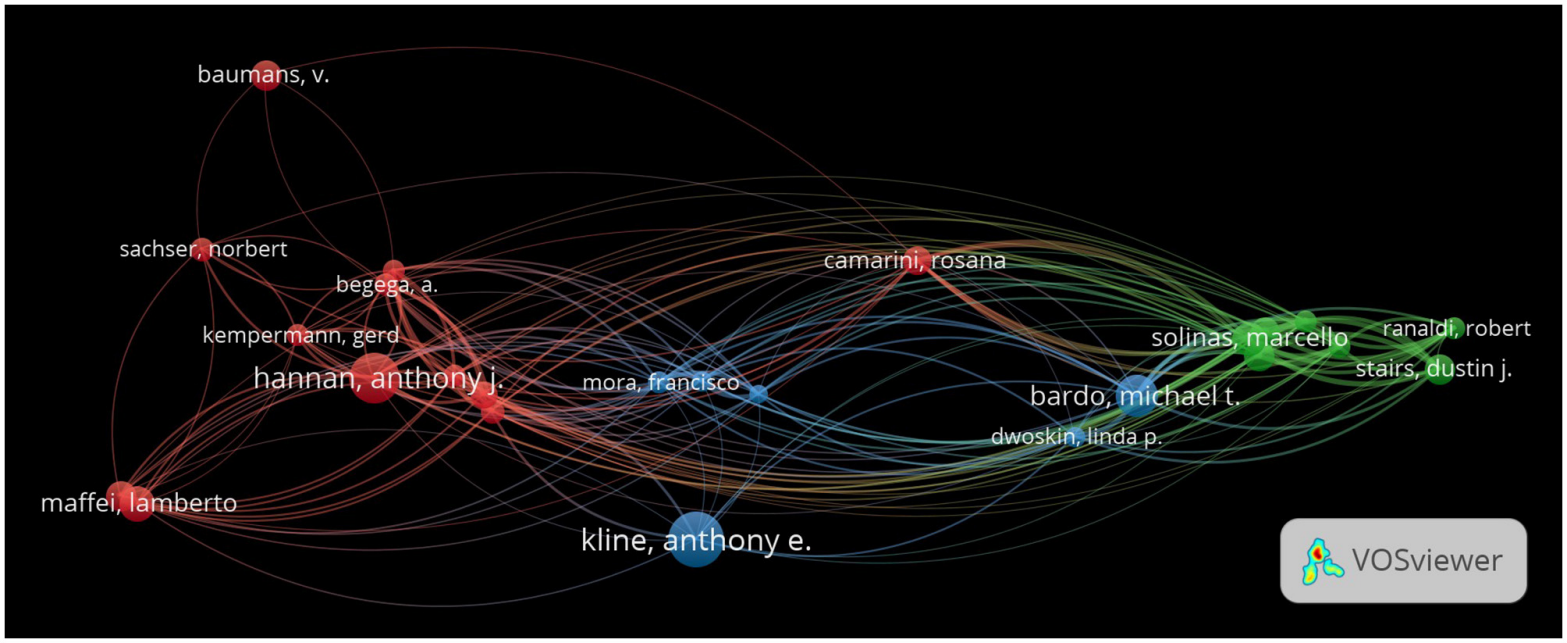
Author collaboration network in EE research. The network visualization shows the collaboration patterns between authors in the field of EE research, with nodes representing individual authors and edges representing co-authorship links. The size of each node corresponds to the number of publications by that author, while the color represents different clusters of collaborative groups. The thickness of the edges represents the strength of collaborations, with thicker lines indicating more frequent co-authorships between authors.

The VOSviewer visualization of author collaborations in EE research illustrates a vibrant network of scholars contributing to the field (Figure 5). The analysis identifies several key clusters of authors, each distinguished by different colors, representing collaborative groups that have significantly impacted EE studies. In this network, nodes represent individual authors, with the size of each node corresponding to the number of publications by that author. Edges or lines between the nodes represent co-authorships, indicating collaborations between authors. The thickness of the lines reflects the strength of the collaboration, where thicker lines represent frequent co-authorship or closer collaborative relationships.

The network is divided into several clusters, each represented by a different color, indicating groups of authors who frequently collaborate within a particular subfield of EE research. For example, red clusters signify a group of authors working on neuroplasticity and neurorehabilitation, including figures such as Anthony J. Hannan, Gerd Kempermann, and Lamberto Maffei. These authors often co-author papers related to neurogenesis, brain plasticity, and the role of EE in mental health, indicating a shared research focus. Green clusters represent another significant group, primarily focused on behavioral outcomes of EE, such as drug addiction and cognitive recovery. Marcello Solinas, Michael T. Bardo, and their collaborators are central figures in this group, as reflected by their relatively large nodes and thick connecting edges, indicating frequent and strong collaborations in this research area. Blue clusters focus on neuroprotection and the effects of EE on recovery from neurological disorders, with authors such as Anthony E. Kline and Francisco Mora playing pivotal roles. The proximity and thickness of the connecting lines between these authors reflect their interdisciplinary collaborations in fields like traumatic brain injury and neuroprotection, showcasing how different themes within EE research overlap and interact.

Anthony E. Kline has emerged as a pivotal figure, represented by the largest node, indicating his extensive collaborations and influence within the EE community. His work primarily connects various aspects of neurorehabilitation and neuroprotection, showing a strong interdisciplinary approach. Michael T. Bardo and Marcello Solinas represent major clusters focused on the behavioral sciences, exploring the implications of EE on drug addiction and cognitive functions. Lamberto Maffei and his collaborators have significantly contributed to understanding the neuroplasticity and sensory processing alterations induced by EE. Anthony J. Hannan has robust ties with his collaborators, particularly around genetic models and mental health outcomes, underscores the translational impact of EE research.

### Author co-citation analysis

In the comprehensive bibliometric analysis on EE, a total of 129,389 author names appeared across the analyzed articles. With a stringent threshold set at a minimum of 200 co-citations (two publications are cited together by a third publication), 70 authors met this criterion. Link strength in this context represents the number of times two authors are co-cited together, indicating how frequently their works are referenced together in the same publication. The top 25 co-cited authors were selected for a more detailed visualization to highlight their contributions and the intricate network of intellectual collaboration within the field (Table 4).

**Table 4:** Top 25 authors based on the number of co-citations.

**Author**	**Co-citations**	**Total link strength**
Kempermann G.	1,458	24,178
Gage F.H.	1,393	23,972
Hannan A.J.	1,059	15,346
Rosenzweig M.R.	899	19,381
Bardo M.T.	795	11,284
Van Praag H.	753	12,973
Kline A.E.	686	20,561
Bennett E.L.	633	15,158
Mohammed A.H.	583	11,357
Greenough W.T.	544	11,372
Maffei L.	529	10,748
Jaber M.	501	10,038
Solinas M.	494	9,448
Nithianantharajah J.	483	8,488
Diamond M.C.	478	12,141
Winblad B.	404	7,834
Kuhn H.G.	397	8,256
Wurbel H.	395	4,694
Berardi N.	380	8,381
Sale A.	374	7,842
Rampon C.	349	6,685
Thiriet N.	332	6,835
Cheng J.P.	323	12,017
Dixon C.E.	312	12,636
Zafonte R.D.	235	9,927

Sorted in order of total number of co-citations, from highest to lowest.

Gerd Kempermann leads with 1,458 co-citations, underscoring his significant impact on studies related to neurogenesis and brain plasticity. His high link strength reflects the frequent co-citation of his work with other influential authors, signifying a central position in the intellectual network of EE research. Following closely is Fred H. Gage, with 1,393 co-citations, renowned for his work in similar domains. The strong link strength between Gage and other top authors indicates that his research is often referenced in conjunction with other prominent studies in the field, illustrating his intellectual influence. Anthony J. Hannan and Mark R. Rosenzweig, with 1,059 and 899 co-citations respectively, also emerge as key figures, particularly noted for their research on cognitive and neural plasticity influenced by environmental factors. The link strength of their co-citations highlights the close thematic relationship of their work with other major contributors in EE research, showing how their studies are frequently grouped with those of other leading figures when cited by the broader scientific community.

This analysis sheds light on the collaborative and intellectual relationships that shape the EE research field. The visualized network based on co-citation patterns reveals which authors works are most often studied together, providing insights into how foundational research clusters have formed and evolved over time.

### Author co-citation network visualization

**Figure 6:**
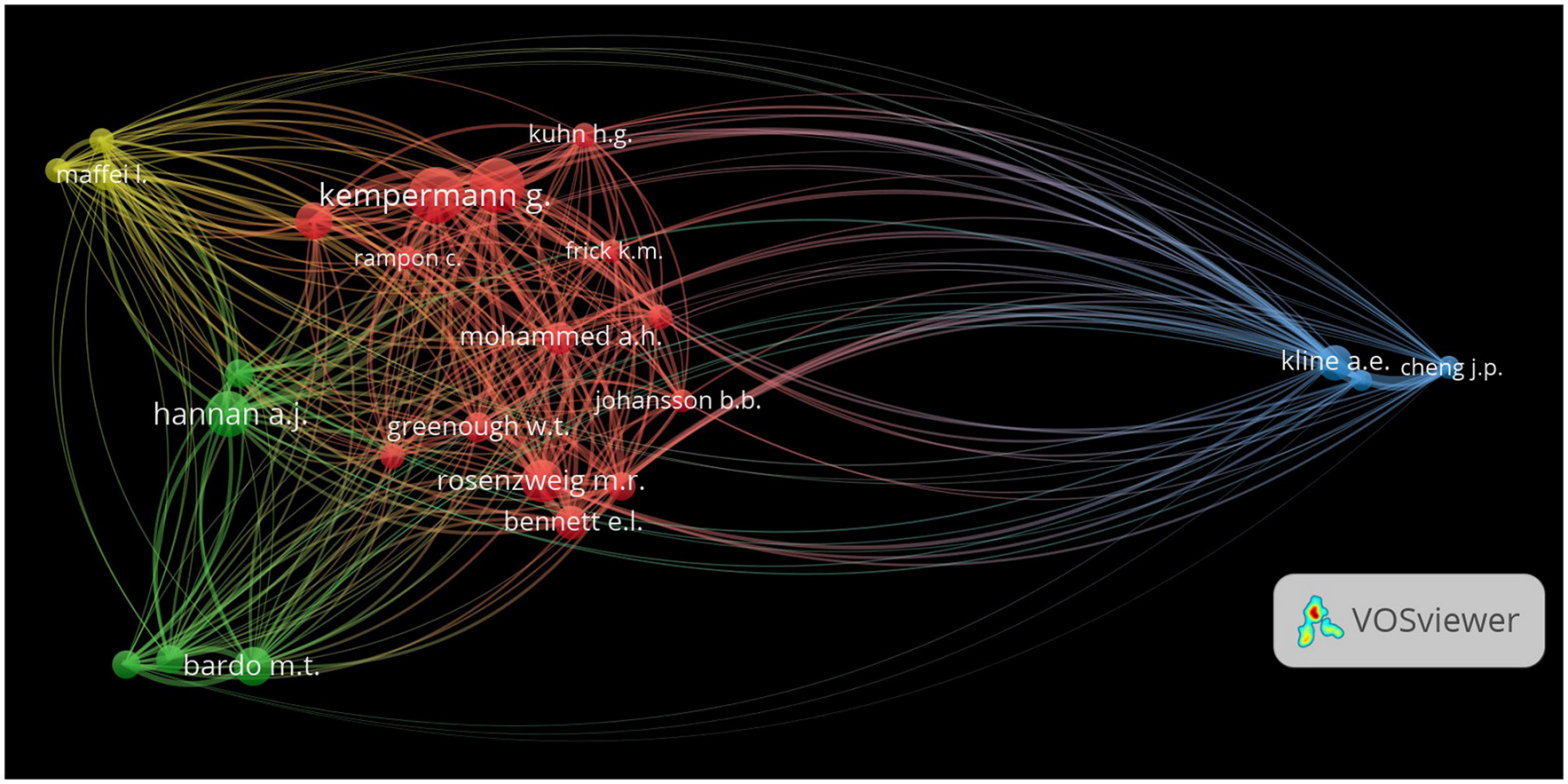
Co-citation network of authors in EE research. The network visualization depicts co-citation relationships between authors frequently cited together in EE research. Nodes represent individual authors, with node size corresponding to the number of times an author has been co-cited, and edges indicating the frequency of co-citations between authors. The thickness of the edges represents the strength of co-citation ties, demonstrating intellectual connections and thematic overlap between authors.

For visualization purposes, the top 25 most co-cited authors were selected, representing the scholarly impact and network connections in EE research. In the co-citation network, nodes represent individual authors, and the size of each node reflects the number of co-citations associated with that author. Larger nodes, such as those representing Anthony E. Kline and Gerd Kempermann, indicate authors who have been frequently co-cited in the literature, marking them as central figures in EE research. Edges or lines between the nodes represent co-citations between authors, where thicker edges indicate more frequent co-citations, suggesting stronger conceptual or thematic links between the works of those authors. The visualization through VOSviewer revealed a dense network of co-citations among leading researchers, with several notable nodes indicating key figures (Figure 6).

Anthony E. Kline and Jeffrey P. Cheng, positioned on the extreme right of visualization, formed a prominent blue cluster, indicating their pivotal roles and frequent co-citations in the literature on neural rehabilitation and neuroplastic effects of EE. The thickness of the connecting lines reflects their strong associations with other researchers, showcasing their influence in shaping this field. Similarly, Gerd Kempermann and Hans G. Kuhn, central in the network and marked in red, illustrate strong interlinkages, primarily related to their work on adult neurogenesis and cognitive enhancement through EE. The red cluster illustrates a tightly knit group of researchers focusing on neuroplasticity and its applications, with robust internal connections that highlight frequent co-citation patterns. Michael T. Bardo and Anthony J. Hannan, shown in green, connect widely with multiple clusters, underscoring their interdisciplinary impacts on behavioral sciences and neurogenetic aspects of EE. The thick lines linking their nodes to other clusters emphasize their research cross-disciplinary relevance, as their work on behavioral outcomes and genetic models of EE is frequently co-cited with studies from various fields. Lamberto Maffei, highlighted in yellow, shows extensive connectivity, emphasizing his contributions to sensory plasticity and neuronal development studies within the context of EE. His position within the network suggests strong ties to other influential researchers, further demonstrating the breadth of his impact.

This analysis highlights the interconnected nature of research on EE. It shows that the original works by highly cited authors facilitate cross-disciplinary dialogues and advancements. This visualization depicts how the research on EE is interwoven, encompassing studies ranging from investigations of molecular mechanisms to therapeutic applications.

### Countries collaboration analysis

During the bibliometric analysis of global collaborations in EE research, contributions from 85 countries were identified. Out of these, at least five publications on EE were recorded from 42 countries, reflecting broad international interest in this field. Link strength in this context measures the degree of collaboration between countries, based on co-authorships across different nations. For the purposes of visualization, the focus was placed on the top 25 most active countries based on their publication output, citation count with other countries, and total link strengths (Table 5). A higher link strength between countries indicates frequent collaboration on shared research projects, showing how closely countries work together in EE research.

**Table 5:** Top 25 countries based on the number of documents, citations, and total link strength from collaboration work with other countries.

**Country**	**Documents**	**Citations**	**Total link strength**
United States	595	29,975	153
United Kingdom	172	8,668	95
Brazil	164	2,487	42
China	116	2,132	35
Canada	109	6,089	48
Spain	105	4,024	55
Australia	98	3,824	41
Germany	92	5,230	43
Italy	87	4,277	38
France	79	5,134	54
Netherlands	72	3,517	56
Japan	52	1,203	19
Sweden	41	3,480	37
Mexico	35	629	12
Switzerland	28	1,938	29
India	26	368	14
Argentina	24	698	11
Denmark	23	938	22
South Korea	21	347	12
Poland	18	517	12
Belgium	17	656	21
Norway	16	552	19
Austria	15	338	30
Portugal	9	443	10
Hong Kong	8	190	10

The United States led the field with 595 documents and a remarkable citation count of 29,975, highlighting its central role in EE research globally. The high link strength of the U.S. with other countries demonstrates its extensive collaborative efforts in driving EE studies forward, as many U.S. researchers frequently co-author with international colleagues. Other highly active countries included the United Kingdom and Brazil, which also showed strong link strengths with various international partners, illustrating their role in fostering global collaborations. Substantial contributions were also noted from countries like Canada, Spain, and Australia as well. The strong collaborative ties between these countries and others in the field have helped in the development and dissemination of EE-related knowledge. The link strength values help highlight the interconnectedness of the global research community, showing which countries have established strong international research partnerships and how these collaborations contribute to the advancement of EE studies.

### Countries collaboration network visualization

**Figure 7:**
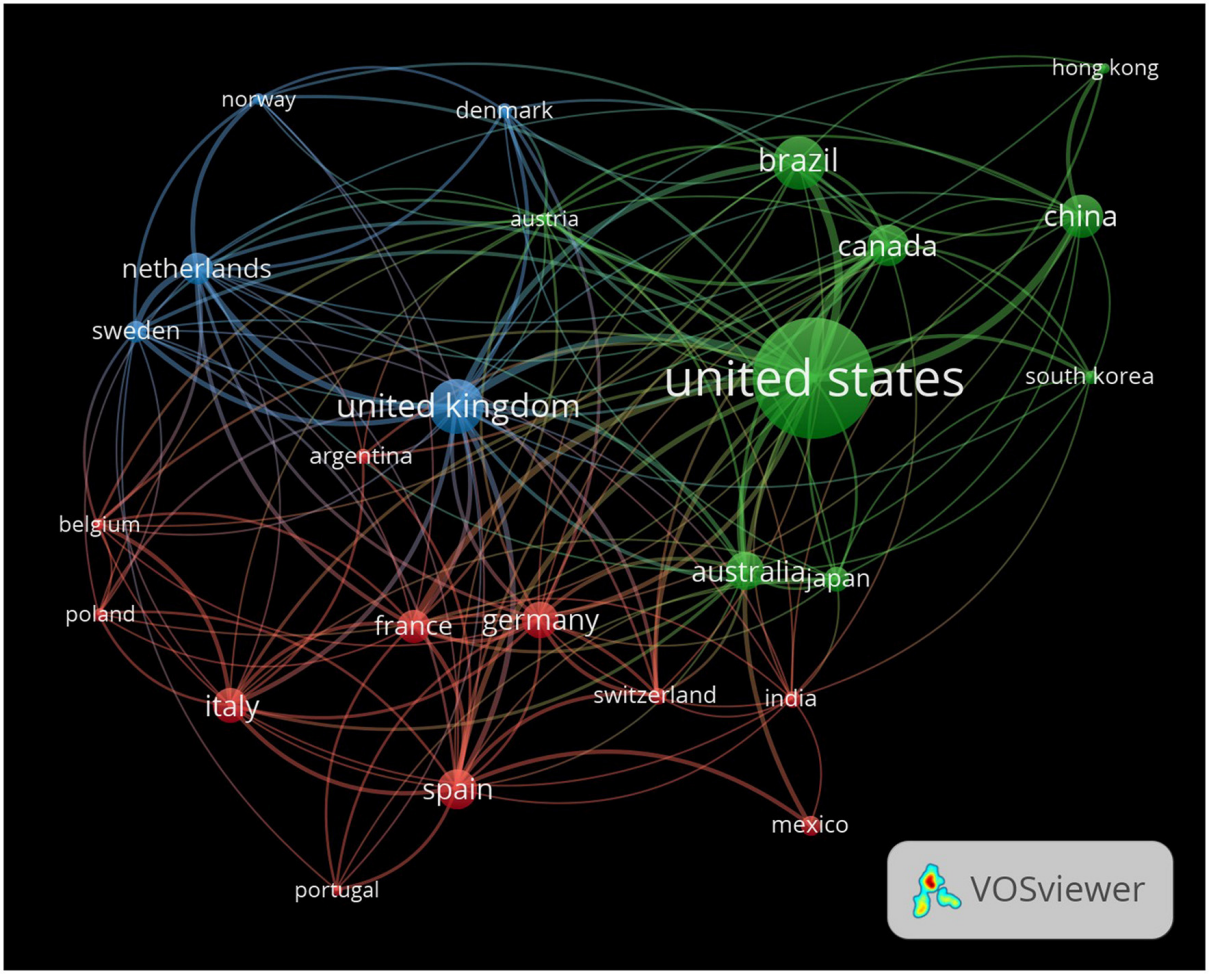
Global collaboration network in EE research. The network visualization illustrates the global collaboration patterns in EE research, with each node representing a country and the edges representing collaborative ties between countries. The size of the nodes corresponds to the number of publications from each country, and the thickness of the edges reflects the strength of collaboration between countries.

The VOSviewer visualization shows global collaboration in EE research (Figure 7). Nodes in the visualization represent individual countries, with the size of each node reflecting the volume of publications produced by that country. Larger nodes, such as the United States, indicate that the country plays a central role in EE research, contributing a substantial number of publications and fostering international collaborations. The edges or lines between nodes represent collaborative relationships between countries, with the thickness of the edges indicating the strength of the collaboration. Thicker lines between two countries, such as the United States and Canada or the United Kingdom, demonstrate frequent co-authorship and strong bilateral research ties, showing how these nations collaborate closely in EE research. The color of the nodes represents different clusters, or groups of countries, that frequently collaborate within specific geographic or thematic areas of EE research.

The green cluster encompasses countries like the United States, China, Brazil, and Canada. This group represents a significant portion of EE research, with the United States acting as a hub that connects these countries through numerous collaborations. The blue cluster features countries like the United Kingdom, Sweden, and the Netherlands, highlighting a group of European nations that work closely together in EE research. The red cluster includes Germany, France, Italy, and Spain, showcasing strong intra-European collaborations. The dense network of connections between these countries indicates frequent research partnerships, suggesting that EE research within Europe is highly interconnected. The proximity of these nodes to one another and the thickness of the lines further underscore their robust research ties, highlighting Europe's role as a collaborative region within the global EE research community. Countries such as South Korea, Brazil, and Australia, represented in various colors, also demonstrate active involvement in EE research. Their strong connections with countries in other clusters, including the United States and China, reflect meaningful partnerships that enhance the global diversity of the research community. While their nodes may be slightly smaller, indicating fewer total publications, the thickness of their connecting lines emphasizes the significance of their collaborations. Additionally, nodes like Japan and India show connections with several key research hubs, indicating their growing participation in global EE research. These connections help integrate diverse geographical regions into the collaborative network, contributing to the advancement of EE studies worldwide.

This visualization illustrates the geographic diversity and interconnected nature of EE research. It highlights the central role of the United States as a global leader, while also showcasing the strong research networks within Europe and between various regions such as North America, Asia, and Latin America. By visualizing these collaborations, the map underscores how international partnerships contribute to the growth and dissemination of knowledge in the field of EE.

### Analysis of journals that published manuscripts on EE

The results of the bibliometric analysis for the top 25 journals published articles on EE revealed a robust and diverse scholarly output (Table 6). Link strength in this context refers to the frequency of co-citation between journals, representing the degree to which journals are cited together within the same research articles. Journals with higher link strength are more frequently co-cited with others, indicating strong intellectual connections between the studies they publish.

**Table 6:** Top 25 scientific journals, determined by the number of documents, citations, and total link strength related to EE research.

**Source**	**Documents**	**Citations**	**Total link strength**
Applied Animal Behaviour Science	100	6,311	162
Behavioural Brain Research	94	4,559	520
European Journal of Neuroscience	23	2,765	208
Journal of Neuroscience	17	2,489	53
Physiology and Behavior	40	1,933	177
Neuroscience	37	1,852	213
PLoS ONE	42	1,534	104
Pharmacology Biochemistry and Behavior	32	1,443	168
Experimental Neurology	25	1,416	45
Zoo Biology	34	1,384	57
Neurobiology of Learning and Memory	21	1,294	120
Proceedings of the National Academy of Sciences of the United States of America	6	1,101	100
Laboratory Animals	16	1,000	46
Brain Research	35	985	145
Psychopharmacology	19	946	117
Neuropharmacology	16	821	91
Neuropsychopharmacology	7	812	98
Neuroscience letters	23	782	81
Frontiers in Behavioral Neuroscience	28	616	122
Animal Welfare	26	557	59
Molecular Psychiatry	5	528	60
Developmental Psychobiology	14	499	58
Psychoneuroendocrinology	10	432	83
Animals	46	398	57
Brain Research Bulletin	17	351	82

The journal *Applied Animal Behavior Science* leads with the highest number of documents (100) and a significant citation count (6,311), highlighting its significant role in the dissemination of EE research, particularly in applied animal behavior contexts. The strong link strength between this journal and others in the network reflects the interdisciplinary nature of EE studies, as its research is often cited alongside work published in other fields. This is closely followed by *Behavioral Brain Research*, which has published 94 documents and received 4,559 citations, demonstrating its significant impact on behavioral studies of EE. The link strength between *Behavioral Brain Research* and other journals emphasizes its importance in connecting behavioral and neurological research, showing frequent co-citation with journals focused on neuroscience. The *European Journal of Neuroscience* and *Journal of Neuroscience* are also prominent in the field and maintain considerable citation impact and frequent co-citation with other journals, particularly in studies exploring neural plasticity and cognitive function related to EE. *Physiology and Behavior* and *Neuroscience* both contribute significantly as well, focusing on physiological responses to environmental factors and their link strengths indicate their role in bridging the gap between physiology and behavior in EE research.

*PLoS One* stands out for its broad scope as it supports interdisciplinary reach and open access to EE research. It has published 42 documents related to EE, facilitating wider dissemination and impact of work on EE. Specialized journals like *Zoo Biology* and *Laboratory Animals* also reflect the application of EE paradigm across fields like zoology and veterinary sciences, indicating the multidisciplinary nature of EE studies.

The journals *Brain Research* and *Experimental Neurology* show focused contributions to understanding the neurological underpinnings and experimental aspects of EE. These journals also have strong link strengths with others in the field, particularly those that focus on neuroplasticity and cognitive studies, indicating that their research is often cited together with key studies in EE. The presence of high-impact journals like *Proceedings of the National Academy of Sciences of the United States of America* (PNAS) with fewer documents but substantial citation numbers highlight key groundbreaking research contributions to the field of EE from the journal. The strong link strength of PNAS indicates that its publications on EE are frequently cited alongside foundational studies in the field, illustrating its influence in shaping major research trends.

### Journals network visualization

**Figure 8:**
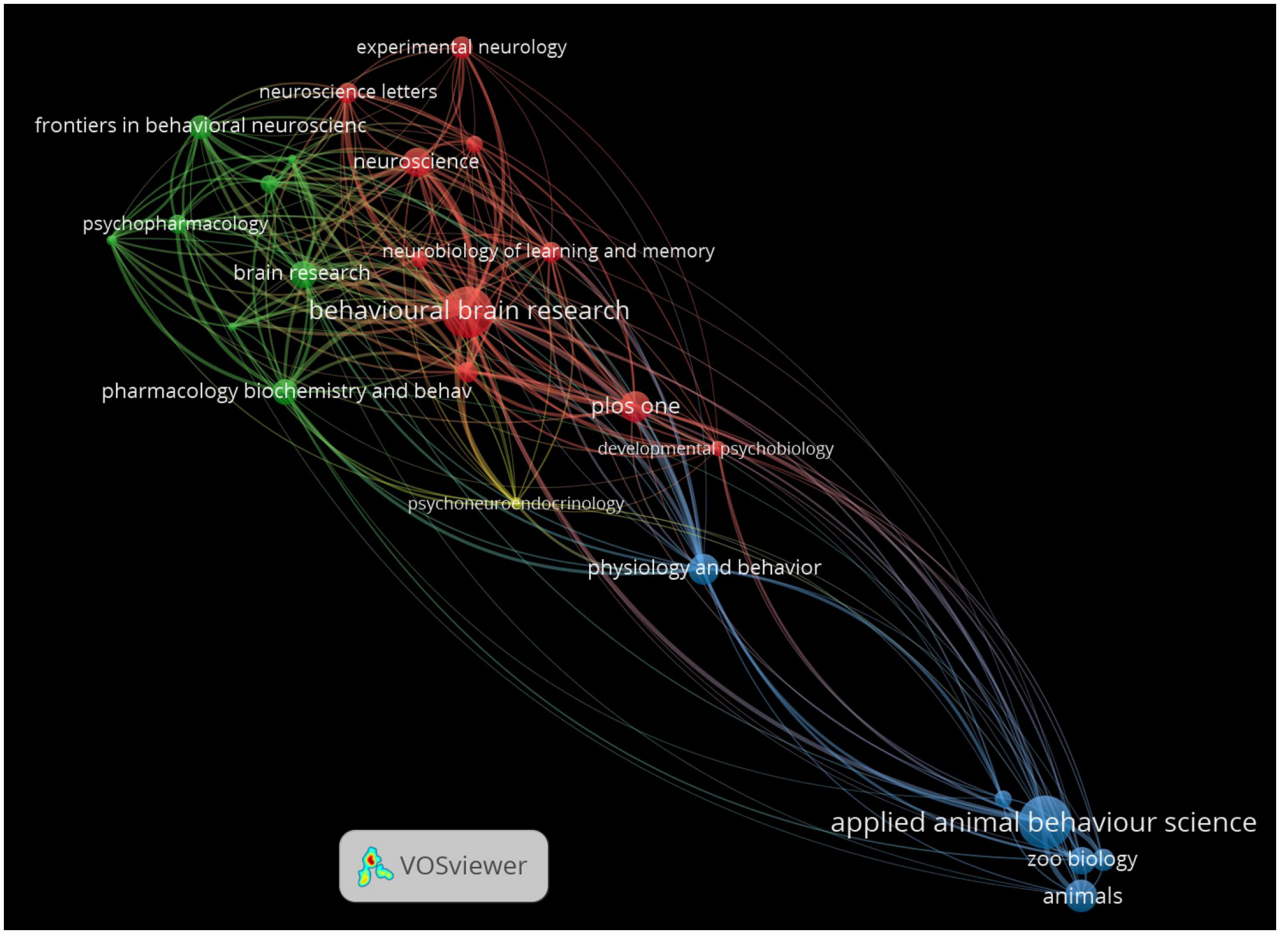
Journal citation network in EE research. The network visualization shows the citation relationships between journals publishing EE research. Each node represents a journal, with the size of the node corresponding to the number of articles published on EE, and the edges representing citation links between journals. The color of each node reflects clusters of closely related journals in terms of their citation patterns.

The visualization of journal citation networks within the field of EE reveals a concentrated interaction among key scientific publications, each playing a significant role in disseminating research on this topic (Figure 8). Nodes in the visualization represent individual journals, and the size of each node corresponds to the number of citations that journal has received within the EE research network. Larger nodes, such as those representing *Behavioural Brain Research* and *Neuroscience*, indicate that these journals are central to EE research and frequently cited by others in the field, reflecting their prominence in publishing influential studies. Edges or lines between the nodes represent citation links between journals, where the thickness of the lines reflects the strength of the citation relationships. For example, the thicker lines between *Behavioural Brain Research, Neuroscience*, and *Brain Research* highlight their close citation relationships and their central role in advancing the neurobiological aspects of EE.

The color-coded clusters represent groups of journals that frequently cite each other, indicating thematic or disciplinary closeness. Journals such as *Behavioural Brain Research, Neurobiology of Learning and Memory*, and *Neuroscience* form a red cluster, highlighting their tight-knit focus on neurological and behavioral research in EE. This cluster demonstrates a strong focus on cognitive function, neural plasticity, and behavioral outcomes of EE, emphasizing their role in contributing foundational research in these areas. The green cluster, containing journals like *Pharmacology Biochemistry and Behavior, Psychopharmacology*, and *Frontiers in Behavioral Neuroscience*, reflects an emphasis on psychopharmacology and behavioral neuroscience, where EE research intersects with studies on drug effects and behavioral outcomes. The frequent citation patterns among these journals illustrate their shared interest in how environmental factors and pharmacological agents influence behavior and brain function.

Journals such as *PLoS One* and *Physiology and Behavior* serve as central connectors, with blue clusters representing their interdisciplinary impact. These journals bridge research areas across neuroscience, physiology, and behavioral sciences, as indicated by their multiple connections to other clusters. The diversity of research covered in these journals makes them key hubs for disseminating a wide range of EE-related studies, from neurobiology to behavior and physiology. Their position in the network underscores the interdisciplinary nature of EE research, which spans multiple fields, including psychoneuroendocrinology, as represented by journals like *Psychoneuroendocrinology*.

Additionally, specialized journals such as *Applied Animal Behaviour Science* and *Zoo Biology* form part of the light blue cluster, representing the extension of EE research into applied animal behavior and zoological studies. Their presence in the network highlights how EE research is not confined to laboratory neuroscience but also plays a role in understanding animal welfare and behavioral enrichment in applied settings, such as zoos and laboratories.

This network visualization highlights the interconnected nature of journal publications within EE research, demonstrating how journals from diverse disciplines contribute to the broader understanding of how environmental factors influence behavior and brain function. The various clusters and the thickness of the connecting lines emphasize the intellectual connections that shape the field and point to the interdisciplinary nature of EE research, spanning core neuroscience, behavior, pharmacology, and applied animal studies.

## Discussion

EE involves enhancing the living conditions of captive animals to promote their physical and psychological wellbeing (Singhal et al., 2014). Since the 1960s, research in this field has evolved significantly, reflecting shifts in scientific focus, technological advancements, and a deeper understanding of neurobiology and animal welfare. This manuscript examined the emerging patterns and trends in EE research from 1967 to 2024 utilizing robust software, such as R and VOSviewer, for bibliometric analysis and to generate co-occurrence networks.

### Early focus on enrichment vs. deprivation (1967–1979)

In the late 1960s and 1970s, early research by Vogel et al. (1967, 1968), Walsh et al. (1972), and Gardner et al. (1975) primarily focused on comparing EE with deprivation. In 1967, keywords like EE, deprivation, and cognitive functioning indicate a focus on how enriched environments affect cognition when compared to deprived settings, signaling a foundational understanding that environmental factors significantly shape brain development and behavior (see Appendix 2 for keywords each year). However, by 1970, research began incorporating specific behavioral tests. Keywords such as open field, food competition behavior, mouse, and running wheel suggest that researchers started measuring the exploratory and feeding behavior in rodents in response to EE, while also suggesting an early interest in the relationship between physical exercise and brain function (Monosevitz, 1970). The use of rodents in these studies reflected a growing trend toward controlled measurable experiments that could provide deeper insights into the behavioral changes brought about by different environmental conditions.

In 1972 and 1973, terms like Hebb-Williams maze, defecation, exploration, and open-field activity further indicate the use of mazes and open-field tests to assess learning and anxiety-related behaviors in rodents reared in enriched vs. standard environments (Smith, 1972; Manosevitz and Montemayor, 1972; Manosevitz and Joel, 1973). Running wheel activity remained a common measure of enrichment. The keyword post-weaning enrichment underscores research exploring the effects of EE at developmental stages, with findings suggesting that EE may help mitigate the impact of early deprivation (Smith, 1972).

In 1975, studies investigated the effects of factors like cage size and environmental conditions on learning, memory, and physiological responses like body weight and water intake (Huck and Price, 1975; Manosevitz and Pryor, 1975). The impact of environmental impoverishment became a central focus, leading to a deeper understanding of how different degrees of environmental stimulation can influence cognitive and physical health. Studies on surface texture as an enrichment factor demonstrated that even seemingly minor changes in the environment could have profound effects on learning outcomes (Manosevitz and Pryor, 1975). By 1976, the scope broadened to examine the effects of environmental crowding on aggression and hormonal responses, highlighting a growing awareness of the social and hormonal dynamics influenced by EE (Hull et al., 1976). In 1977, a new avenue of research emerged, where EE was studied in the context of recovery from brain injuries or lesions. Researchers explored how brief periods of enrichment following post-weaning cerebral lesions could promote recovery of learning and memory in rodents, particularly in animals exposed to methamphetamine (Will, 1977). This year marked the beginning of using EE as a potential therapeutic tool for brain recovery, laying the groundwork for later studies on neuroplasticity and rehabilitation.

By 1978, studies began incorporating pharmacological compounds, as seen in the use of keywords like chlorpromazine and strychnine (Cummins et al., 1978). This prompted investigation on how EE interacts with neurochemicals related to arousal and activity. The exploration of drug-environment interactions indicated an emerging complexity in the understanding of how external stimuli, environmental and chemical, could combine to affect brain and behavior. Overall, the early period represents a transition in EE research, from behavioral observations to the exploration of social, developmental, and pharmacological factors, setting the stage for the more mechanistic and molecular investigations that emerged in the following decades.

### Emergence of neural mechanisms (1980–1989)

The 1980s marked a shift toward exploring the neural effects of EE. In 1981, research began to explore how EE could influence neurodevelopment under challenging conditions. For example, studies looked at the effects of prenatal x-irradiation and microcephaly in conjunction with EE (Shibagaki et al., 1981; Kiyono et al., 1981). The focus on benzodiazepine receptors and tasks like the Hebb-Williams maze suggested that EE was being studied not only in relation to behavior but also in how it could impact specific neuroreceptors and cognitive outcomes in rodents. The combination of prenatal damage and EE interventions reflected an interest in how early-life environmental factors could shape later developmental trajectories. By 1982, the scope broadened to examine how EE influenced exploratory behavior, maze learning, and reactivity, particularly under conditions of environmental restriction and septal lesions (Engellenner et al., 1982). Researchers investigated how EE or its restriction affect both behavior and the brain structure, emphasizing the role of EE in mitigating the negative effects of brain lesions or environmental deficits. In 1983, a shift toward investigating the neurophysiological processes underlying the effects of EE was observed. Studies on cortical development, active sleep, and clonidine administration in infant rats indicated that EE was now being examined for its influence on developmental neuroplasticity and the interactions between environmental stimuli and pharmacological agents (Mirmiran and Uylings, 1983; Mirmiran et al., 1983).

By 1985, the keyword prenatal EE suggests that research had expanded to investigate how enrichment during prenatal development could influence outcomes like maze learning, pointing to an interest in the developmental origins of cognitive effects (Kiyono et al., 1985). In 1986, the use of terms like 6-hydroxydopamine and acetylcholine indicates that researchers began delving into specific neurochemical systems, such as dopamine and acetylcholine pathways, to understand how EE affects neurotransmitter functioning (Dunnett et al., 1986). The involvement of brain regions such as the fimbria-fornix, hippocampus, nucleus basalis magnocellularis, and septum indicated that EE was being investigated for its impact on learning, memory and attention-related pathways through these neurotransmitter systems.

In 1987 and 1988, the focus expanded to recovery from brain injuries. The use of keywords like compensation, contralateral neglect, hemidecorticate rat, and recovery by Rose et al. (1987, 1988) and Rose (1988) suggest their focus on investigating how EE helps in rehabilitating cognitive and motor functions after cerebral damage. The use of recovery and compensation as keywords suggests that EE was seen as a mechanism by which animals could adapt and recover functions that had been impaired by damage to the brain or nervous system. Behavioral assessments of learning and memory continued to play a central role, as indicated by the consistent use of the Hebb-Williams maze and discrimination reversal (Rose et al., 1988; Venable et al., 1988). This period also saw research on Macaca mulatta, signaling that the research had expanded beyond rodents to primates, further reinforcing the generalizability of findings related to EE across species (Reinhardt et al., 1988; Ross and Everitt, 1988).

By 1989, research became more focused on the cellular and anatomical effects of EE. Key terms such as basal dendrite, Golgi method, occipital cortex, and pyramidal neuron indicated that EE started being examined for its influence on dendritic morphology and neural connectivity (Venable et al., 1989). The emphasis on ethanol intake and its relationship to environmental conditions suggested that researchers were also beginning to explore how EE could interact with substance use and addictive behaviors (Rockman et al., 1989). Overall, the 1980s marked a crucial transition from examining behavioral outcomes to exploring the neural underpinnings of EE, particularly in the context of how enriched environments could affect brain structure, neurotransmitter systems, and compensatory mechanisms following neurological damage or deprivation. This decade laid the groundwork for the molecular and cellular studies of brain plasticity and repair that dominated EE research in the following years.

### Social behavior and stress (1990–1999)

During the 1990s, there was increased emphasis on the interplay between stress, abnormal behaviors, and neurobiology in animal models. Early research in the 1990s primarily concentrated on the behavioral effects of EE, particularly in non-human primates, such as Macaca mulatta (Champoux et al., 1990; Line et al., 1990). Keywords such as abnormal, behavior, cortisol, serotonin, and stress reflected an increased focus on how EE influence the physiological stress response, particularly through the modulation of hormonal and neurotransmitter systems (Line et al., 1990; Widman et al., 1992; Carlstead and Shepherdson, 1994; Klein et al., 1994). Keywords from 1991, such as cage furnishings, further emphasize how environmental modifications like enriched cages affected animal wellbeing (Carlstead et al., 1991). Studies explored how EE could mitigate the negative effects of stress and promote better welfare for laboratory animals, as indicated by terms like single caging, object enrichment, and environment (Champoux et al., 1990). The studies in this early period also began to delve into cognitive recovery, with tasks like the Morris water maze being used to study learning and recovery of function, particularly in rodent models (Wainwright et al., 1994).

By the mid-1990s, there was a noticeable shift in the subjects of EE studies from primates to rodents. Researchers began to investigate the social aspect of EE. Keywords such as breeding colony, maternal behavior, aggression, and social restriction in 1995 highlighted a growing interest in the role of early experiences and social interactions in shaping behavior and development (Schapiro et al., 1995). Simultaneously, the neurophysiological mechanisms behind these behaviors were increasingly explored. Studies began to investigate corticosterone, testosterone, nerve growth factor, and fatty acid metabolism to understand how EE impacted brain structure and function (Barnard et al., 1996; Pham et al., 1999; Van de Weerd et al., 1997). Cognitive tasks like the Hebb-Williams maze and Lashley maze continued to feature prominently, emphasizing the growing recognition of the role of EE in enhancing cognitive performance and mitigating stress-related behaviors in laboratory animals (Boehm et al., 1996).

As EE research progressed into the late 1990s, the focus became more molecular, with an emphasis on understanding the neuroplastic and neurochemical mechanisms underlying the effects of enrichment. The keywords from 1998 and 1999 indicate a significant interest in glutamate receptors, NGF mRNA, and astrocytes, suggesting that researchers were increasingly investigating how EE affected brain plasticity at the cellular level (Torasdotter et al., 1998; Gagné et al., 1998; Soffi et al., 1999). At the same time, the species studied became more diverse, with EE research extending beyond rodents and primates to include dogs, turtles, guinea pigs, rabbits, and turkeys (Dean, 1999; Lidfors, 1997; Crowe and Forbes, 1999; Sherwin et al., 1999a,b; Burghardt et al., 1996). This expanded interest in applying EE paradigms across a variety of contexts and understanding its broader biological implications, such as the effects of EE on pecking behavior. Research on EE also started including other primates, such as apes and chimpanzees (Wood, 1998). There was also an increasing focus on welfare and ethical concerns, as terms like psychological wellbeing and regulatory toxicology became more prevalent in the literature (Dean, 1999; Burghardt et al., 1996; Wood, 1998; Boinski et al., 1999).

Overall, the decade from 1990 to 1999 saw a marked progression from addressing behavioral and stress responses in isolated or restricted environments to exploring the complex molecular, neurophysiological, and ethical dimensions of EE. The inclusion of some keywords, such as amphetamine, dopamine, and nucleus accumbens, by a group of scientists points to their focus on the neurochemical mechanisms underlying social behaviors and reward systems (Bowling et al., 1993; Bardo et al., 1995, 1999). The growing interest in neuroplasticity, neuronal health, and the molecular pathways that are influenced by EE helped researchers understand how enriched environments could promote better cognitive function, stress resilience, and overall wellbeing across various species. Overall, this decade saw a substantial shift toward understanding how EE influences both the social and neurochemical aspects of animal behavior, setting the stage for deeper molecular investigations in the 2000s, particularly in the context of mental health, neurobiological changes, and neurodevelopmental disorders, and thereby opened new avenues for both scientific inquiry and animal welfare practices.

### Neurotrophic factors and neurogenesis (2000–2009)

In the decade between 2000 and 2009, EE research expanded significantly, with increasing focus on its effects on animal welfare, neuroplasticity, neurogenesis, neurodegeneration, behavior, and neurochemical processes across various animal models, including rodents, fish, pigs, rabbits, parrots, bats, and primates. Throughout the decade, keywords like neurotrophins, BDNF, and fibroblast growth factor indicate that studies were increasingly exploring how EE influences the expression of neurotrophic factors critical for synaptic plasticity and neuron growth (Ickes et al., 2000; Turner and Lewis, 2003; Will et al., 2004; Rossi et al., 2006; Angelucci et al., 2009).

At the beginning of the decade, research on EE placed a strong emphasis on animal welfare, particularly examining the effects of enriched environments on pigs. These studies highlighted improvements in learning, behavior, stress reduction, and circadian rhythm regulation under enriched condition (Beattie et al., 2000a,b; de Jong et al., 2000; Sneddon et al., 2000). Simultaneously, the cognitive and behavioral implications of EE were being explored extensively in rodent models, with a focus on enhanced memory, spatial learning, visual acuity, contextual processing, and stress resilience (Woodcock and Richardson, 2000; Varty et al., 2000; Prusky et al., 2000). This period also marked the initial investigations into the potential of EE to combat neurodegenerative diseases such as Alzheimer's and Parkinson's, with research demonstrating its role in reducing oxidative stress and improving cognitive performance. Key areas of focus included mitochondrial health and the impact of caloric intake on the progression of these diseases (Mattson et al., 2001).

As the decade progressed, studies delved deeper into the cellular mechanisms underlying the effects of EE, such as neuroplasticity and emotional reactivity, and addressed behaviors linked to addiction, social environments, and traumatic brain injury (Duffy et al., 2001; Hoplight et al., 2001; Passineau et al., 2001; Williams et al., 2001; Bardo et al., 2001). Rodent studies expanded to explore a wider range of behaviors, including anxiety, learning, novelty seeking, and exploration, while also underscoring the influence of EE on neurogenesis, dendritic spine density, and cellular markers like cytochrome oxidase (Fernández-Teruel et al., 2002a,b; Schrijver et al., 2002; Komitova et al., 2002; Johansson and Belichenko, 2002). EE research also began to include developmental and acquired conditions, such as traumatic brain injury and Down syndrome, suggesting its growing recognition as a potential therapeutic intervention for both genetic and environmental brain abnormalities (Hicks et al., 2002; Wagner et al., 2002; Martínez-Cué et al., 2002). The importance of social interaction was emphasized through comparisons of single and social housing models, illustrating how social components enhance the effects of EE (Schapiro, 2002). Similarly, the connection between EE and stress regulation became more prominent, with a focus on the modulation of the HPA axis and reductions in corticosterone levels. These findings emphasized the role of EE in mitigating physiological and behavioral stress responses (Belz et al., 2003; Morley-Fletcher et al., 2003). Additionally, research investigated how EE could influence immune functions, play behavior, and repetitive behaviors in transgenic mouse models, expanding understanding of its broad neuroendocrine and immunological impact (Marashi et al., 2003). Alzheimer's disease research remained a focal point, with studies revealing how EE reduces amyloid plaque buildup, enhances synaptic plasticity, and promotes neurogenesis through neurotrophins like BDNF, thereby supporting overall hippocampal health (Turner and Lewis, 2003; Jankowsky et al., 2003).

Around the middle of the decade, EE research placed more emphasis on neurotrophic factors like BDNF and fibroblast growth factor, linking them to synaptic plasticity and memory consolidation (Will et al., 2004). Researchers were increasingly interested in how enriched environments could modulate long-term potentiation, gene activation, and cellular processes like dendritic branching and synapse regulation (Keyvani et al., 2004; Cancedda et al., 2004; Johansson, 2004; Nithianantharajah et al., 2004). Researchers continued to focus on neurogenesis, synaptic plasticity, and the role of EE in conditions like traumatic brain injury and Alzheimer's disease (Bruel-Jungerman et al., 2005; Gaulke et al., 2005; Percaccio et al., 2005; Wagner et al., 2005; Jankowsky et al., 2005; Lambert et al., 2005). The introduction of auditory stimulation (Percaccio et al., 2005; Lutz and Novak, 2005) and epigenetic factors into EE research marked a new direction, indicating an interest in how environmental factors interact with sensory and genetic mechanisms to affect brain function (Friske and Gammie, 2005). EE research expanded further to include autism models (Schneider et al., 2006), and explored how enriched environments might influence behaviors related to stereotypy, anticipatory behaviors, and functional recovery after spinal cord injury (Schneider et al., 2006; Wood et al., 2006; Erschbamer et al., 2006). Researchers also examined how chronic stress impacted the effects of EE, particularly in relation to neurogenesis and HPA-axis regulation (Welberg et al., 2006). By 2007, the focus on brain plasticity became even more pronounced, with studies examining neurogenesis, synaptophysin levels, and the role of neurotransmitters like acetylcholine and dopamine in enriched environments (Mora et al., 2007; Niu et al., 2007; Fan et al., 2007; Leal-Galicia et al., 2007; Arco et al., 2007). The role of EE in recovery from brain injury, spinal cord injury, and ischemic stroke was a growing area of interest, highlighting its potential therapeutic applications (Berrocal et al., 2007; Pereira et al., 2007; Buchhold et al., 2007). Themes like behavioral synchronization (Scott et al., 2007) and anxiety-related behavior (Leal-Galicia et al., 2007; Iwata et al., 2007) were explored reflecting a broader consideration of welfare in enriched environments.

Toward the end of the decade, EE studies continued to focus on neurogenesis and synaptic transmission, with research incorporating neurotrophic factors, addiction models, and stress-related behaviors (Kondo et al., 2008; Parks et al., 2008; Rahman and Bardo, 2008; Brenes and Fornaguera, 2008; Qian et al., 2008; Segovia et al., 2008; Solinas et al., 2008). An increasing attention was given to the impact of EE on conditions like stroke (Plane et al., 2008; Wang et al., 2008), depression (Brenes and Fornaguera, 2008), Alzheimer's disease (Görtz et al., 2008; Herring et al., 2008), and schizophrenia (McOmish et al., 2008). Research also began to examine gene-environment interactions, exploring how EE could influence gene expression in response to stress, exercise, and neurodegenerative and neurodevelopmental conditions (Kondo et al., 2008; Herring et al., 2008; McOmish et al., 2008; Bernberg et al., 2008; Stam et al., 2008; Thiriet et al., 2008). There was also a growing interest in the role of EE in addiction, particularly how it could modulate drug-seeking behavior and relapse (Rahman and Bardo, 2008; Solinas et al., 2008; Smith et al., 2008; Grimm et al., 2008). By 2009, EE research had become highly diverse, exploring topics such as neurodegenerative diseases (Catlow et al., 2009; Herring et al., 2009; Mirochnic et al., 2009), addiction (Chauvet et al., 2009; El Rawas et al., 2009; Solinas et al., 2009; Thiel et al., 2009; Stairs and Bardo, 2009), and developmental plasticity (Cai et al., 2009; Caleo et al., 2009; Petrosini et al., 2009). Research emphasized the cellular effects of EE, focusing on dendritic spines (Goshen et al., 2009; Gelfo et al., 2009; Fréchette et al., 2009), synaptic reorganization (Caleo et al., 2009), and neuroplasticity (Darmopil et al., 2009). The role of EE in addiction models, including studies on drug self-administration and protracted abstinence, reflected an increasing interest in how environmental factors could modulate behavioral and physiological responses to drugs (Chauvet et al., 2009; Stairs and Bardo, 2009; Smith et al., 2009). The integration of advanced techniques like *in vivo* microdialysis (El Rawas et al., 2009), Magnetic Resonance Imaging (MRI) (Nag et al., 2009), and molecular biology methods, such as immunohistochemistry (Herring et al., 2009) provided a mechanistic understanding of how EE impacts neural circuits involved in various brain functions, offering new insights into its potential therapeutic applications.

### Behavioral and molecular studies (2010–2019)

Between 2010 and 2019, research on EE expanded significantly each year and focused on both the molecular and genetic mechanisms by which EE influences brain function. This underscores the importance of discussing the progression of EE research on a year-by-year basis.

In 2010, studies highlighted the roles of BDNF, cortisol, dopamine, and other neurochemicals, which are critical for neural plasticity, stress regulation, and emotional wellbeing, in relation to EE (Sun et al., 2010; Lonetti et al., 2010; Segovia et al., 2010; Munsterhjelm et al., 2010; Zajac et al., 2010). EE was shown to significantly improve spatial memory, reduce anxiety, and enhance cognition, with outcomes often linked to changes in neurotransmitter systems and neural circuit modulation (Sun et al., 2010; Nowakowska et al., 2010; Chen et al., 2010; Hughes and Collins, 2010; Sztainberg et al., 2010). The Morris Water Maze and Y-maze tasks assessed cognitive behavior, revealing substantial improvements in spatial learning and memory in enriched animals (Chen et al., 2010; Sparling et al., 2010). Additionally, the antidepressant effects of EE were explored using behavioral assays like the forced swim test, indicating its potential to alleviate depressive-like symptoms (Green et al., 2010). Notably, EE was explored in brain conditions such as Alzheimer's disease (Herring et al., 2010), drug addiction (Solinas et al., 2010), Rett syndrome (Lonetti et al., 2010; Kerr et al., 2010), and Huntington's disease (Wood et al., 2010; Benn et al., 2010). Rodent models remained central to this research, demonstrating how EE paradigms influence key brain areas like the hippocampus, nucleus accumbens, amygdala, frontal cortex, and striatum (Sun et al., 2010; Segovia et al., 2010; Zajac et al., 2010; Sztainberg et al., 2010; Mainardi et al., 2010). Advanced techniques such as gene expression profiling (including microarray and real-time RT-PCR), neuroimmune responses and neuroimaging were increasingly used to investigate the underlying molecular mechanisms that contribute to EE-induced changes in behaviors and brain plasticity (Kerr et al., 2010; Benn et al., 2010; Workman et al., 2010; Devonshire et al., 2010; Arranz et al., 2010). EE was also studied in non-rodent species, such as pigs and marsupials, to understand its impact on behavioral and physiological outcomes (Munsterhjelm et al., 2010; Oostindjer et al., 2010; Hogan et al., 2010). The interplay between EE, stress, and neural plasticity emerged as a critical theme (Hendriksen et al., 2010; Schloesser et al., 2010). The exploration of EE extended to complex neurobehavioral paradigms, such as incentive sensitization and conditioned place preference, which investigated its role in addiction and reward processing (Green et al., 2010). Techniques like *in vivo* imaging provided insights into how EE modulates neurogenesis, synaptogenesis, and neurotransmitter levels (Segovia et al., 2010; Mainardi et al., 2010). The growing use of transgenic and knockout mice, combined with a focus on transcriptional and epigenetic regulation, paved the way for a deeper understanding of how EE could be harnessed to develop therapeutic interventions for various neurological and psychiatric conditions (Zajac et al., 2010; Herring et al., 2010; Wood et al., 2010; Benn et al., 2010).

In 2011, EE research broadened considerably, emphasizing its effects on neurogenesis, neuroprotection, and recovery from neurological and psychiatric disorders, as well as stress-related conditions. Studies highlighted the role of EE in key brain regions, such as the hippocampus, amygdala, and prefrontal cortex, which are critical for learning, memory, executive functions, and emotional regulation (Munetsuna et al., 2011; Peng et al., 2011; Rawas et al., 2011; Wooters et al., 2011). EE interventions were shown to mitigate anxiety, aggression, and stereotypic behaviors by reshaping neural circuits (Kim and Sufka, 2011; Akre et al., 2011; Melotti et al., 2011). Research increasingly focused on neurodegenerative diseases, like Alzheimer's (Arranz et al., 2011; Rodríguez et al., 2011), and emphasized the ability of EE to alleviate prenatal stress (Peng et al., 2011), boost functional recovery (Zai et al., 2011), and promote social behavior (Workman et al., 2011) and neural resilience (Lehmann and Herkenham, 2011). EE also proved beneficial in neurodevelopmental disorders, with Down syndrome models, such as Ts65Dn mice, showing enhanced neural regeneration and cognitive improvement (Baamonde et al., 2011; Begenisic et al., 2011). Furthermore, the impact of EE on neuroinflammatory conditions was linked to decreased microglial activity (Lahiani-Cohen et al., 2011), and its influence on dopamine system underscored its effects on reward and motivational pathways (Wooters et al., 2011). Beyond rodents, EE improved welfare and cognitive function in zoo species. For instance, captive animals, including chimpanzees and parrots, exhibited reduced stress behaviors and heightened adaptability through the use of toys and structured spaces (de Andrade and de Azevedo, 2011; Zaragoza et al., 2011). Physical exercise integrated with EE was found to support neuronal survival and growth (Chakrabarti et al., 2011). On a molecular level, techniques like real-time RT-PCR (Munetsuna et al., 2011) and immunohistochemistry (Bednarek and Caroni, 2011; Lanosa et al., 2011) revealed that EE modulates neurogenesis and synaptogenesis markers, while epigenetic research confirmed its potential to induce lasting gene expression changes, providing a robust framework for long-term brain health and resilience (Lopez-Atalaya et al., 2011).

By 2012, EE research had evolved to incorporate a diverse range of molecular techniques, deepening the understanding of its neurobiological effects. Studies began to examine glial cell roles, specifically microglia and astrocytes, and the involvement of immune proteins, such as cytokines and chemokines (Williamson et al., 2012; Jurgens and Johnson, 2012). In Alzheimer's models, EE showed promising results in reducing amyloid-beta deposition and memory deficit (Maesako et al., 2012). Likewise, EE emerged as a potential intervention for traumatic brain injury, demonstrating efficacy in promoting functional recovery and reducing neuronal loss (Cheng et al., 2012; Kline et al., 2012). The research into stress hormones, including corticosterone and HPA axis regulation, explored the role of EE in alleviating physiological stress, with direct implications for treating anxiety and aggression (Du et al., 2012; Skwara et al., 2012; Hutchinson et al., 2012a,b). The hippocampus and prefrontal cortex remained a focal area, with findings that EE upregulates neurotrophins like BDNF, enhances synaptic connectivity, and improves learning and memory (Chourbaji et al., 2012; Rueda et al., 2012; Kumar et al., 2012; Zhang et al., 2012). EE was also linked to reductions in stereotypic and abnormal behaviors, underscoring its significance in improving animal wellbeing (Gross et al., 2012; Meagher and Mason, 2012). EE research expanded to explore aging, emphasizing its ability to combat cognitive decline and oxidative damage (Lee et al., 2012). EE also displayed potential in reducing drug-seeking behavior and preventing relapses, with research pointing to the modulation of dopamine system as central to these effects (Alvers et al., 2012; Gomez et al., 2012; Klaissle et al., 2012). Additionally, an *in vivo* study significantly advanced the understanding of how EE fosters brain plasticity and facilitates recovery processes (Buschler and Manahan-Vaughan, 2012).

In 2013, the role of EE on hippocampal neurogenesis was extensively studied, revealing how enriched conditions can stimulate the proliferation of neural progenitor cells and enhance dendritic spine density that translates into improved learning and memory (Pons-Espinal et al., 2013; Speisman et al., 2013; Tanti et al., 2013). EE was shown to mitigate age-related cognitive deficits, suggesting its potential to counteract the decline in neuroplasticity associated with aging and neurodegenerative diseases, such as Alzheimer's disease (Speisman et al., 2013; Barak et al., 2013; Beauquis et al., 2013). EE was shown to lower corticosterone levels (Garrido et al., 2013), modulate HPA axis (Sampedro-Piquero et al., 2013), and reduce anxiety-like behaviors in paradigms such as the elevated plus maze (Ravenelle et al., 2013) and elevated zero-maze (Sampedro-Piquero et al., 2013). Neurotransmitter systems, including dopamine, serotonin, and acetylcholine, were investigated to understand the role of EE in influencing emotional and cognitive processes. The regulation of dopamine receptors (Zeeb et al., 2013) and serotonin pathways (Renoir et al., 2013) contributed to the effects of EE on reward, motivation, and emotionality related behaviors. The role of cholinergic signaling in improving spatial learning and memory was also explored (Harati et al., 2013). EE was shown to mitigate symptoms and improve social and cognitive outcomes in models of autism (Reynolds et al., 2013) and research on genetic factors, such as Dyrk1a in Down syndrome models, provided insights into the gene-by-environment interactions that influence neurodevelopment and behavior (Pons-Espinal et al., 2013). EE influence on seasonal epigenetic regulation, specifically through histone variant H2A.Z, revealed a dynamic interplay between environmental factors and chromatin modifications that affect gene expression (Simonet et al., 2013). Furthermore, a systematic review introduced the concept of enviromimetics, or the use of environmental cues to mimic the benefits of physical exercise and cognitive stimulation (Pang and Hannan, 2013).

In 2014, the hippocampus remained a primary area of focus, given its critical role in learning, memory, and emotional regulation (Barichello et al., 2014; Morelli et al., 2014). The involvement of glucocorticoid receptors and the modulation of neuroimmune responses, such as reductions in cytokine levels, were explored to understand neuroprotective effects (Connors et al., 2014; Durairaj and Koilmani, 2014; Gurfein et al., 2014; Sampedro-Piquero et al., 2014). In Alzheimer's models like the App23 and 3 × Tg-AD mice, EE reduced amyloid-β accumulation and improved cognitive function, shedding light on the potential of non-pharmacological interventions to slow disease progression (Polito et al., 2014; Blázquez et al., 2014). The use of advanced techniques such as two-photon *in vivo* imaging and proteomics provided insights into the cellular and molecular changes induced by EE (Jung and Herms, 2014; Lichti et al., 2014). The continued study of neurotransmitter systems, including dopamine, serotonin, and acetylcholine, revealed how EE influences mood, motivation, and cognitive processes (Hofford et al., 2014; Batzina et al., 2014a,b; Lima et al., 2014). Models of drug addiction, including methamphetamine and nicotine sensitization, demonstrated that EE reduced drug-seeking behavior and altered neurochemical reactivity (Hofford et al., 2014; Hamilton et al., 2014). EE was shown to influence glial cell activation, promoting a shift from a pro-inflammatory to an anti-inflammatory phenotype. Microglial polarization, and by changes in cytokine expression such as TNF-α and IL-1β, contributed to a healthier neural environment (Pusic et al., 2014; Briones et al., 2014). Mechanisms such as DNA methylation were linked to experience-dependent plasticity, offering a deeper understanding of how environmental factors improve learning and memory in aged animals (Irier et al., 2014). The role of EE in autophagy and metabolism was also examined, with studies suggesting that enriched conditions may promote brain health through the regulation of cellular homeostasis and energy balance (Takahashi et al., 2014). Furthermore, the modulation of synaptic and structural plasticity in regions like the prefrontal cortex and nucleus accumbens was crucial for understanding the potential of EE in treating cognitive and emotional dysfunctions (Lichti et al., 2014; Fan et al., 2014; Mychasiuk et al., 2014). The use of EE in a model of epilepsy highlighted its impact on neuronal excitability and network stability, suggesting avenues for therapeutic applications (Morelli et al., 2014).

In 2015, the hippocampus remained a focal point, with studies highlighting the role of EE in enhancing neurogenesis, increasing synaptic plasticity, and improving learning and memory (Ahmadalipour et al., 2015; Morse et al., 2015; Soares et al., 2015; Valero-Aracama et al., 2015). BDNF upregulation was associated with increased hippocampal cell proliferation and dendritic spine density, supporting the idea that EE promotes brain plasticity and cognitive recovery (Ahmadalipour et al., 2015; Mosaferi et al., 2015; Novkovic et al., 2015). The modulation of signaling pathways, such as the extracellularly regulated kinase (ERK) and cAMP response element-binding protein (CREB), were found to link molecular changes to functional outcomes (Gomez et al., 2015; Matteucci et al., 2015). EE altered reward-related behaviors and reduced relapse rates in models of drug addiction, including methamphetamine and heroin dependence (Hajheidari et al., 2015a,b; Peck et al., 2015). Enrichment interventions, such as sensory and physical enrichment, were explored to reduce stereotypic behaviors like feather-pecking in birds (Clyvia et al., 2015; Hartcher et al., 2015). Studies on prenatal and early postnatal stress underscored the protective effects of EE, with enriched conditions mitigating the long-term impact of adverse experiences (Pascual et al., 2015; Zubedat et al., 2015). Similarly, sensory and social stimulation attenuated the age-related decline in the mRNA expression of hippocampal steroidogenic enzymes (Rossetti et al., 2015). The intersection of EE and physical activity was also explored, with studies demonstrating that combining exercise with cognitive enrichment amplifies the benefits on brain plasticity and overall wellbeing. This was particularly relevant for aging populations, where EE and exercise were shown to prevent cognitive decline and promote resilience against age-related diseases (Torres-Lista and Giménez-Llort, 2015). Model of social isolation highlighted the detrimental effects of deprivation, which could be reversed through enriched rearing conditions (Tanaś et al., 2015). Social interaction and positive reinforcement training in domesticated animals, such as cats and goats, were found to improve behavior and welfare (Oliveira et al., 2015; Rehnberg et al., 2015).

In 2016, the hippocampus and HPA axis continued to be the focal areas, as EE was shown to enhance neurogenesis, promote synaptic plasticity, reduce anxiety and improve memory and learning (Stein et al., 2016; Catuara-Solarz et al., 2016; Kapgal et al., 2016; Ronzoni et al., 2016). Studies examining early life stress and prenatal stress exposure found that EE could reverse or mitigate the negative effects on brain development and behavior, highlighting its therapeutic potential (do Prado et al., 2016; Rajesh et al., 2016). The use of paradigms like conditioned place preference provided insights into how EE can modulate reward pathways and prevent relapse (Mustroph et al., 2016). EE strategies like foraging enrichment were explored to improve welfare and reduce stereotypic behaviors (Coelho et al., 2016). A study showed that the benefits of EE could extend across generations, with offspring of enriched parents exhibiting better stress resilience, cognitive performance, and social behavior (Li et al., 2016). Dietary supplements, such as epigallocatechin-3-gallate and anthocyanins, were also studied in conjunction with EE, revealing potential synergistic effects on brain health and neuroprotection (Catuara-Solarz et al., 2016; Kreilaus et al., 2016). The use of techniques like high-throughput sequencing, transcriptomics, and proteomics provided insights into the molecular pathways involved in EE-induced recovery (Dowle et al., 2016; Zhang et al., 2016a,b).

In 2017, the hippocampus remained central to the EE research like previous years and the role of EE in alleviating anxiety, depression, and stress was extensively studied. Research into emotional wellbeing emphasized the role of glucocorticoid receptors and the epigenetic regulation of stress responses, with findings suggesting that EE could have long-term protective effects on mental health (Novaes et al., 2017; Shilpa et al., 2017; Yeshurun et al., 2017). In models of Alzheimer's disease, EE reduced amyloid accumulation and improved cognitive function (Salmin et al., 2017), while in models of traumatic brain injury, EE promoted functional recovery, and neural regeneration (Liu et al., 2017). Studies on addiction highlighted the role of EE in reducing drug-seeking behavior and craving, particularly for substances like cocaine, methamphetamine, and opioids (Gauthier et al., 2017; Hajheidari et al., 2017; Hofford et al., 2017). Paradigms such as cue-induced reinstatement provided insights into how EE can modulate reward pathways, offering potential therapeutic avenues for treating substance use disorders (Gauthier et al., 2017; Glueck et al., 2017). Studies on captive psittacines explored the use of enrichment objects and sensory stimulation to improve welfare and reduce stereotypic behaviors (Carvalho et al., 2017; Prathipa et al., 2017). EE was found to contribute to greater resilience against stress and psychiatric disorders like posttraumatic stress disorder (PTSD) (Novaes et al., 2017; Blanco et al., 2017; Nawaz et al., 2017). The intersection of EE with physical exercise was widely explored, as combining physical exercise with EE was shown to amplify neurogenic and neuroprotective effects (Rajab et al., 2017; Sakalem et al., 2017). The use of acetylcholinesterase inhibitors (de la Tremblaye et al., 2017), dopamine agonists (Glueck et al., 2017), and serotonin transporter (Rogers et al., 2017) in research provided insights into how EE affects synaptic transmission and receptor expression, offering new perspectives for treating psychiatric and neurodegenerative disorders.

In 2018, research on early-life stress, including maternal separation and juvenile isolation, revealed that EE could mitigate the long-term adverse effects on brain development and behavior, underscoring its potential as a non-pharmacological intervention for stress-related disorders (Dandi et al., 2018; Durán-Carabali et al., 2018; Borniger et al., 2018). EE was shown to counteract the negative effects of aging, and oxidative stress (Cintoli et al., 2018; Seo et al., 2018). Studies using paradigms such as conditioned place preference and self-administration showed that EE decreased drug-seeking behavior and craving for substances like alcohol, cocaine, and heroin (Freese et al., 2018; Imperio et al., 2018; Wang et al., 2018). The reduction in addictive behaviors was linked to changes in dopamine signaling and alterations in the nucleus accumbens, a key region involved in reward and motivation (Brown et al., 2018; Grimm et al., 2018). Studies on broiler chickens, pigs, and primates demonstrated that environmental stimulation, such as providing opportunities for natural behaviors, improved welfare and reduced harmful behaviors (Baxter et al., 2018; Chou et al., 2018; Costa et al., 2018). The influence of EE on epigenetic mechanisms and transgenerational inheritance continued to gain attention (Cintoli et al., 2018; Griñán-Ferr et al., 2018; Benito et al., 2018). EE was shown to induce changes in DNA methylation, histone modifications, and microRNA expression, which could be passed on to subsequent generations (Imperio et al., 2018; Benito et al., 2018). Neurodevelopment and early-life interventions were explored to understand the critical windows during which EE has the most significant impact (Durán-Carabali et al., 2018; Bator et al., 2018).

In 2019, research on EE continued to shed light on its substantial impact on behavior, brain function, and overall welfare, with a focus on both rodent models and practical applications for animals in agricultural and zoological settings. EE was shown to provide protection against the negative effects of chronic stress, adolescent stress, and early-life adversity, enhancing resilience and emotional regulation (González-Pardo et al., 2019a; Dixon and Hughes, 2019; Moradi-Kor et al., 2019). Research in rodent models showed significant reductions in anxiety- and depressive-like behaviors when exposed to EE, attributed to the upregulation of neurotrophic factors like BDNF and reductions in neuroinflammation (Singhal et al., 2019a,b; Wang et al., 2019). The role of glucocorticoids and serotonin in mediating the effects of EE on mood and behavior was also explored (Tatemoto et al., 2019; Lopes et al., 2019). The use of controlled cortical impact models demonstrated that EE could improve motor function, reduce brain tissue loss, and enhance synaptic connectivity (Besagar et al., 2019; Bleimeister et al., 2019; Lajud et al., 2019). In agricultural animals, such as broiler chickens, EE strategies that included straw provision, group housing, and sensory enrichment improved welfare, reduced stereotypic behaviors, and enhanced growth and production outcomes (Meyer et al., 2019; Vasdal et al., 2019). In zoo animals, enrichment items and structured activities were implemented to reduce stress, promote natural behaviors, and improve overall wellbeing (Fernandez and Timberlake, 2019; Regaiolli et al., 2019). The role of microglia in mediating neuroinflammatory responses to EE was also investigated, revealing that EE promotes an anti-inflammatory environment conducive to neuroprotection (Ali et al., 2019; Stuart et al., 2019). Studies on oxidative stress emphasized the role of antioxidant enzymes and dietary interventions, such as green tea extract, in enhancing the neuroprotective effects of EE (De Toma et al., 2019). The impact of EE on maternal separation highlighted its role in shaping wellbeing across the lifespan (Doreste-Mendez et al., 2019; González-Pardo et al., 2019b).

In conclusion, across 2010–2019, research into EE expanded significantly and focused on several key areas: neurotrophic factors and brain plasticity, neurogenesis and neuroprotection, stress and cortisol regulation, neurodegenerative diseases and cognitive decline, and spatial memory and learning. In the early part of the decade, keywords such as epigenetic, CREB, and transcription factors indicate a growing interest in how EE modulates gene expression and neural circuitry (Solinas et al., 2010; Lopez-Atalaya et al., 2011; Simonet et al., 2013; Matteucci et al., 2015; Xie et al., 2012). By the mid-2010s, the research expanded into deeper molecular explorations, with terms like microRNA (Ragu Varman et al., 2013; Pusic and Kraig, 2014; Gomez et al., 2016), DNA methylation (Rossetti et al., 2015), histone acetylation (Morse et al., 2015), and neuroinflammation (Jurgens and Johnson, 2012; Aranda et al., 2019) pointing to studies investigating how EE could influence genetic and inflammatory processes within the brain. Additionally, this decade saw an increased focus on psychiatric and neurological disorders, as reflected by increasing use of keywords such as depression, anxiety, addiction, and traumatic brain injury (Stein et al., 2016; Dandi et al., 2018; Singhal et al., 2019a; Rogers et al., 2017; Imperio et al., 2018; Wang et al., 2019; Ragu Varman et al., 2013; Stairs et al., 2016; Gong et al., 2018; Tomiga et al., 2016; Novaes et al., 2018; Alwis et al., 2016; Bondi and Kline, 2017). Behavioral assessments, such as the Morris water maze and elevated plus maze, continued to be used extensively to evaluate cognitive performance and anxiety-related behaviors in animals exposed to EE (Dandi et al., 2018; Rogers et al., 2017; Ragu Varman et al., 2013; Gong et al., 2018; Novaes et al., 2018). This period represents an important shift toward understanding how EE affects the brain on a genetic and molecular level, with growing implications for its use in treating neuropsychiatric and neurodegenerative conditions.

### Recent trends and advanced paradigms (2020–2024)

Analyzing the keywords from 2020 to 2024 reveals several emerging patterns and trends in EE research. This period saw ongoing research interest in brain plasticity, with terms like neurogenesis, synaptic plasticity, axon regeneration, and dentate gyrus increasingly becoming popular in EE studies (Khalil, 2024; Tang, 2020; de Oliveira et al., 2020; Yan et al., 2023). This trend highlights a growing interest in understanding how EE influences brain plasticity, particularly in diseases like Alzheimer's disease (Zhu et al., 2024), and anxiety and depression (Kimura et al., 2022; Wei et al., 2022). BDNF has been a consistent focal point, emphasizing research on how EE engages molecular pathways that promote neuroplasticity (Tan et al., 2021; Costa et al., 2023; Ismail et al., 2024). In particular, the BDNF-TrkB signaling pathway has been studied for its role in underpinning memory and cognition improvements associated with EE (Ismail et al., 2024). Moreover, neuroinflammation and oxidative stress are frequently featured, especially in studies investigating neuroprotection in affective disorders and neurodegenerative diseases (Kimura et al., 2022; Seo et al., 2020).

Behavioral and cognitive enhancements have also remained significant research themes. The role of EE in improving cognitive performance, learning, memory, and behavior is well-documented, with positive outcomes consistently measured through behavioral paradigms like the Barnes maze and elevated plus maze (Heimer-McGinn et al., 2020; Belo-Silva et al., 2024). By 2024, research focused on advanced cognitive functions, as indicated by keywords such as context, episodic memory, object recognition, pattern separation, and place cell (Pintori et al., 2022; Ventura et al., 2024; Riaz et al., 2021; Dickson et al., 2024). These terms suggest that studies were exploring how EE enhances higher-order cognitive processes, including memory and spatial learning. There has been a particular surge in research focused on adolescence and aging, suggesting that EE interventions might offer therapeutic potential across different stages of life, from age-related memory decline to enhancing emotional resilience and supporting developmental milestones (Song et al., 2021; Suárez et al., 2020; Dandi et al., 2022; Brancato et al., 2020).

Research has also expanded to investigate non-cognitive outcomes of EE, including immune functions and metabolic health. Keywords like inflammation, oxidative stress, and mitochondrial function highlight growing interest in the ways EE may improve overall physiological health (Seo et al., 2020; Lee et al., 2023; Durán-Carabali et al., 2024; Dwir et al., 2021; Réus et al., 2023). Emerging research into the gut-brain axis is also notable, as studies increasingly explore how EE could influence gut microbiota and immune responses, particularly in models of stress, aging, and neuroinflammation (Zhu et al., 2024, 2022; Orock et al., 2020; Colombino et al., 2021). The modulation of corticosterone and the regulation of the HPA axis continue to remain central themes, with EE often linked to reduced anxiety behaviors and improved emotional stability (Fan et al., 2021; Muthmainah et al., 2021). There is also a growing body of work on how EE can mitigate the negative impact of early life stress, especially in the context of emotional dysregulation (Borba et al., 2021; Rule et al., 2021; Xu et al., 2022).

The scope of EE research has broadened to include a wide range of species, extending beyond traditional rodent models to encompass swine, poultry, primates, sun bear, sea lions, zebrafish, turtles, dolphins, and marsupials. This expansion reflects an increasing recognition of the benefits of EE for animal welfare in agricultural and zoo settings. Keywords related to enrichment devices (Harvey-Carroll et al., 2023), social enrichment (Lauderdale and Miller, 2020), and physical enrichment (Brunet et al., 2023) are becoming more central, with research emphasizing the reduction of stereotypic behaviors and the overall enhancement of animal wellbeing (Donald et al., 2023; Abdul-Mawah et al., 2022). Furthermore, EE continues to be a prominent topic in studies on neurodevelopmental and neurodegenerative disorders, such as autism spectrum disorder (Queen et al., 2020; Favre et al., 2021) and Down syndrome (Toma et al., 2020; Alemany-González et al., 2022), as well as Alzheimer's disease (Wei et al., 2020; Nam et al., 2024) and Parkinson's disease (Seo et al., 2020; Kim et al., 2021). Research in this area focuses on how enriched environments can mitigate or reverse cognitive deficits, synaptic damage, and behavioral abnormalities, with the use of genetic models like APP/PS1 mice for Alzheimer's (Fulopova et al., 2021) and Ts65Dn mice for Down syndrome (Toma et al., 2020) underscoring the relevance of EE as a non-pharmacological intervention. Studies revealed sex-specific responses to EE (Dickson and Mittleman, 2021; Cosgrove et al., 2022; Marion et al., 2022; Monje-Reyna et al., 2022; Sabzalizadeh et al., 2022; Dandi et al., 2024; Weiner et al., 2024), emphasizing the need for tailored interventions.

Research on EE continued to investigate cellular and molecular pathways, while also expanded to explore more complex cognitive and social behaviors. Keywords such as PTEN, mTOR, and CREB-binding protein shows investigation on how EE affects intricate cellular mechanisms related to brain plasticity, neurogenesis, and synaptic function (Tang, 2020; Huang et al., 2020). The integration of novel technologies, like CRISPR-Cas9, expanded the understanding of EE associated molecular mechanisms (Sandoval-Quintana et al., 2023). Research on DNA methylation, histone acetylation, and microRNAs further reflects the trend of exploring how environmental factors like EE can induce lasting changes in gene expression (Wei et al., 2020; McKibben and Dwivedi, 2021; Arraes et al., 2024; Reiser et al., 2021; Lin et al., 2020).

Additionally, the therapeutic potential of EE has gained attention, particularly in the context of rehabilitation and functional recovery following stroke or traumatic brain injury (Lee et al., 2023; Lin et al., 2020; Zhu et al., 2021; de Boer et al., 2020). Terms like self-administration, addiction, and ventral tegmental area indicate an interest in how EE impacts reward systems and addiction-related behaviors (Nicolas et al., 2022; Galaj et al., 2023; Razavinasab et al., 2022). Simultaneously, the continued use of keywords like welfare, behavior, stress, anxiety, and social housing reflects a sustained commitment to improving animal wellbeing, with a focus on reducing stress and promoting natural behaviors. Overall, the current period features the integration of molecular, cognitive, and welfare facets in EE research, aiming to understand both the underlying biological mechanisms and the practical applications of EE in enhancing mental health and animal welfare.

**Figure 9:**
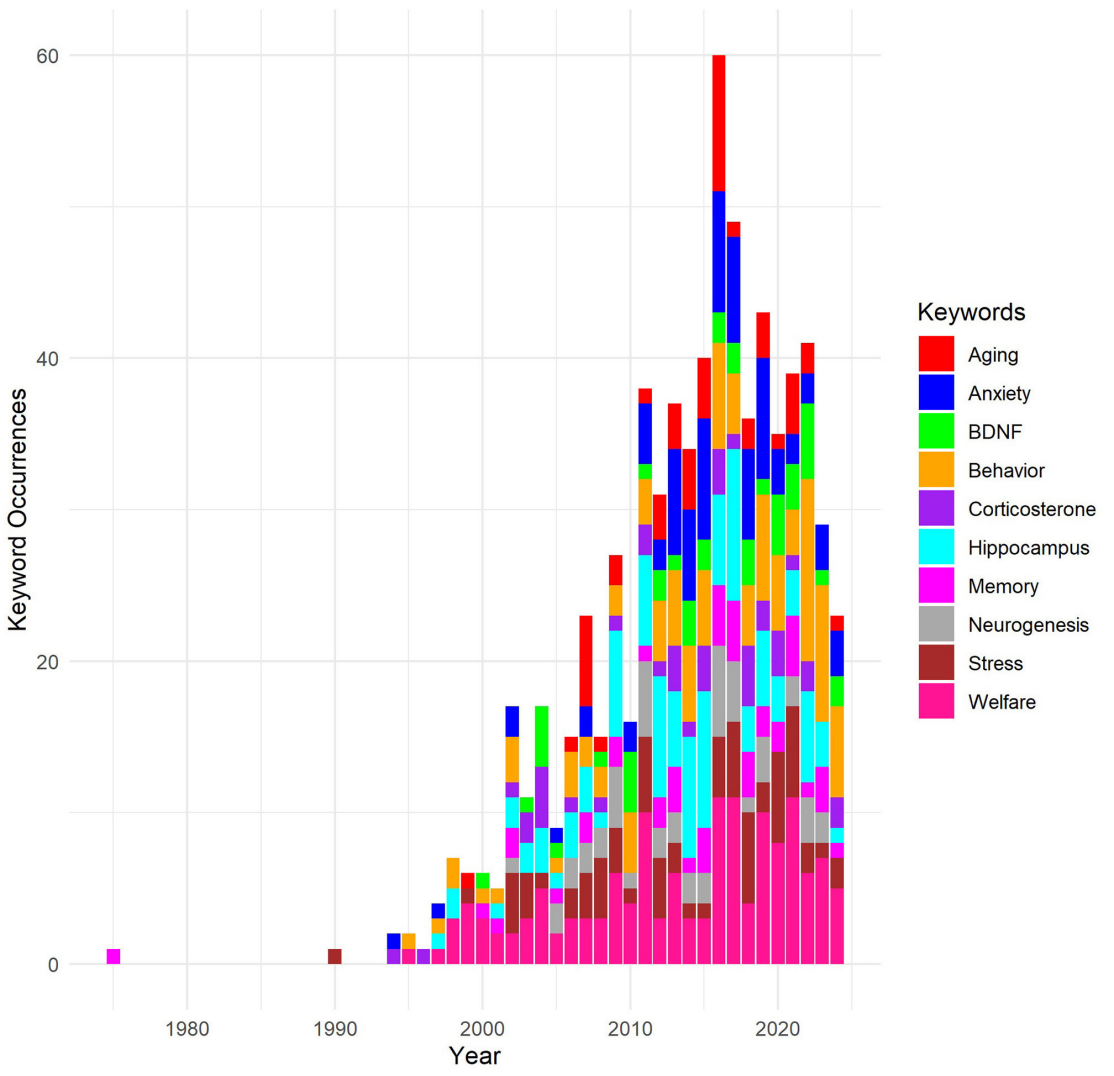
Top 10 Keywords in Environmental Enrichment Research (1967–August 2024). The figure presents a longitudinal analysis of the top 10 keywords associated with EE research from 1967 to August 2024.

Over the past five decades, research on EE has evolved from a simple focus on improving animal welfare in laboratory settings to a complex and multifaceted field. Initially aimed at understanding the effects of enriched environments on brain anatomy and behavior, the field has grown to include studies on neuroplasticity, gene-environment interactions, and the therapeutic potential of EE for neurological and psychiatric disorders. The stacked bar plot of the top 10 keywords in EE research from 1967 to August 2024, as shown in Figure 9, provide valuable insights into the evolving focus of the field. The Y-axis represents the frequency of keyword occurrences per year, with each bar section corresponding to one of the top keywords. This visualization highlights the increasing complexity and diversity of EE research topics over time. In earlier years, keywords such as behavior and welfare appear most prominently, indicating an initial focus on these aspects of EE. As research expanded, keywords like hippocampus, BDNF, and neurogenesis gained prominence, reflecting a shift toward understanding the neurobiological mechanisms of EE, particularly in terms of brain structure and plasticity. The notable occurrence of keywords such as aging, memory, and anxiety after 2010 indicates a growing interest in the therapeutic applications of EE for age-related cognitive decline, memory deficits, and mental health conditions over the past decade. Corticosterone, frequently studied alongside stress, underscores the focus on stress-related biomarkers and their modulation through EE. Overall, the stacked bar plot emphasizes the multidisciplinary nature of EE research, encompassing fields like neuroscience and animal welfare. This trend points toward an expanding scope of EE, with research increasingly focused on molecular mechanisms, neuroplasticity, and translational applications of EE for mental health and age-related brain disorders.

As we move forward, EE research will likely continue to expand into new areas, including its applications for aging populations, neurodegenerative diseases, and mental health disorders. The growing focus on molecular mechanisms and epigenetics suggests that future studies will delve even deeper into how the environment shapes brain functions at the most fundamental levels. EE is not just about improving cognitive and emotional wellbeing in animals but is increasingly recognized as a powerful tool for promoting health across the lifespan, in both animals and humans.

### Influence of key authors

Our bibliometric analysis shows significant contributions from a select group of researchers whose work has shaped the field of EE. These researchers represent diverse continents, highlighting the global interest in EE research. They have contributed to a broad spectrum of topics, ranging from behavioral sciences to neurorehabilitation and neuroplasticity. Notably, Anthony E. Kline emerged as the most central figure, with his extensive collaborative network and a substantial body of work that spans various aspects of EE, particularly in neurorehabilitation and neuroprotection (Bondi and Kline, 2017; Kline et al., 2007; Hoffman et al., 2008). Lamberto Maffei's work also stood out, particularly for his contributions to understanding neuroplasticity and changes in visual sensory processing induced by EE (Rossi et al., 2006; Cancedda et al., 2004; Baroncelli et al., 2013; Bartoletti et al., 2004). His high citation count reflects the substantial impact and relevance of his research in the field. Furthermore, the extensive collaboration network of Anthony J. Hannan indicates a strong focus on the neuropsychiatric implications of EE, linking genetic models with mental health outcomes (Singhal et al., 2019a,b, 2021, 2020). Gerd Kempermann's research, particularly on adult hippocampal neurogenesis (Fabel et al., 2009; Mirochnic et al., 2009; Kempermann et al., 2002), underscores the regenerative and organizational potential of the brain under enriched conditions. Similarly, others, such as Michael T. Bardo and Marcello Solinas, have highlighted the role of EE in addiction and reward system modulation through their research (El Rawas et al., 2009; Hofford et al., 2017; Green et al., 2002; Gipson et al., 2011; Nader et al., 2012).

These key authors not only advance the scientific understanding of EE but also play crucial roles in mentoring the next generation of researchers and shaping future research directions. Their extensive citations and collaborations highlight their influential positions within the scientific community, making their works pivotal in the ongoing research on the applications and benefits of EE.

### Journal contributions to EE research

The role of journals in disseminating EE research is critical. *Applied Animal Behaviour Science* and *Behavioural Brain Research* have been instrumental in bridging experimental research with clinical applications, emphasizing the translational aspect of EE studies. These journals not only lead in terms of document volume but also in influencing the research community, as seen in their citation impacts.

Journals like *European Journal of Neuroscience* and *Journal of Neuroscience* provide platforms for cutting-edge research that connects physiological findings with neurological implications of EE. Their focused contributions are pivotal in driving forward the understanding of how EE affects brain function and structure.

The presence of *PLoS One* highlights the role of open-access journals in enhancing the accessibility and impact of EE research across various scientific disciplines. This broadens the reach of research findings, facilitating cross-disciplinary research that enriches the field. There is also a strong presence of journals such as *Physiology & Behavior* and *Psychopharmacology*, which have contributed to understanding the role of enriched environments modulate hormonal responses, stress physiology, and behavioral outcomes. Additionally, journals like *Developmental Psychobiology* and *Psychoneuroendocrinology* have provided insights into how early-life environmental factors shape long-term neurobiological outcomes. Overall, these journals have collectively advanced the field, ensuring that findings reach a wide and varied scientific audience. This widespread dissemination fosters collaboration, innovation, and a comprehensive understanding of the role, benefits and mechanisms of EE across various species and contexts.

### Global contributions and collaborations

The analysis of contributions by country revealed a diverse and global effort in EE research. The United States leads in both publication volume and citations, reflecting its central role in the global EE research. However, the significant contributions from countries like Brazil, China, Australia and European nations underline a widespread international commitment to advancing EE research. Brazil's prominence in the network points to its active research community focused on animal welfare and neurobiology, while China's increasing contributions underscore its growing influence in neuroscience and behavioral studies. Australia's research efforts are closely related to translational studies that address both human and animal wellbeing. European countries such as the United Kingdom, Germany, Spain, France, and Italy also play major roles, with robust research networks and a strong focus on cross-disciplinary studies. These nations often collaborate with each other, creating a web of research network that facilitate knowledge exchange and innovation.

This geographic diversity is crucial as it introduces a variety of research perspectives and cultural contexts into EE studies, enhancing the generalizability and applicability of research findings. International collaborations, as seen in the co-authorship and citation networks, are vital for sharing resources, knowledge, and techniques, which facilitate advancements in research and practical implementations of EE principles.

### What does the future hold?

The dynamic collaborations among key researchers, influential journals, and global contributions forms a robust framework that supports the continuous growth of EE research. This will ensure sustained progress and innovation in the field, helping to address new challenges and opportunities presented by evolving scientific and technological settings.

By continuing to build on the foundational work of leading authors and leveraging the strengths of top journals and diverse international contributions, the EE research community can enhance its impact on neuroscience, psychology, psychiatry and beyond. The integrative approach will not only advance the understanding of EE but also its practical applications in improving cognitive functions, mental health, and overall wellbeing in various populations.

## Limitations of the study

Our bibliometric analysis of research on EE is extensive and informative, yet there are several limitations that require consideration. For instance, the study relied exclusively on data extracted from Scopus, which, despite its comprehensive coverage, may not include all relevant publications, especially those indexed in other significant databases like PubMed or Web of Science. While we acknowledge this limitation, we decided to rely solely on Scopus for several reasons. Scopus provides a wide range of high-quality peer-reviewed literature across various disciplines, including neuroscience, psychology, and behavioral sciences. This ensured that our analysis captured the core of EE research across multiple fields. Including additional databases, while potentially adding value, would have considerably increased the complexity of the data analysis without proportionally enhancing the quality or scope of the insights derived. By using a single, well-established database, consistency in the data curation process was maintained, avoiding potential redundancies that might arise from merging datasets from multiple sources. Expanding the analysis to multiple databases increases the risk of introducing duplicate entries and errors during data integration. This would require extensive data cleaning to remove duplicates and resolve inconsistencies, which could lead to potential inaccuracies and misinterpretation of the data. Bibliometric analyses rely heavily on the accurate extraction and analysis of metadata (e.g., titles, abstracts, keywords, and citations). Metadata formats can vary significantly across different databases, which could also lead to inconsistencies when merging datasets. Scopus provides well-structured metadata, ensuring uniformity and accuracy in the analysis process. Additionally, with nearly 1,850 articles included, we believe that our dataset is large and comprehensive enough to identify key trends, influential studies, and global contributions to EE research and can reflect patterns and trends on EE research with high accuracy.

It can be imagined that restricting the analysis to articles published in English could have excluded valuable research contributions available in other languages, however, it must be noted that 99.5 % (1,847 out of 1,856) of the indexed articles were published in English so the exclusion of non-English articles did not have any significant impact on the analysis.

We also recognize that citations are not always indicative of quality or scientific value, as they are influenced by various factors, including publication age and the network effect, where well-cited papers continue to attract more citations. Therefore, given the diversity of our dataset, we performed a broader analysis of publication patterns and research themes, which helped us understand the impact beyond mere citation metrics.

The decision to focus on the top 25 results in various categories for visualization purposes might have resulted in the exclusion of relevant but less cited or recently published authors, journals, and countries. It can be said that this approach could limit the representation of emerging trends and contributors who might be playing significant roles but have not yet gained extensive recognition. However, we believe that the primary purpose of bibliometric study is to highlight the most influential and active contributors, providing clear insights into the core areas of research and development, while still noting the potential for emerging trends in the broader discussion.

It is important to note that while our analysis highlights the global nature of EE research, the actual distribution might still be skewed toward institutions and countries with more resources and better access to large research databases. Since we targeted Scopus for our dataset, we could only focus on the publications that Scopus indexes.

## Concluding remarks

This bibliometric analysis of research on EE provides a comprehensive overview of the significant contributions of key researchers, the crucial role of leading journals, and the global impact of studies across numerous countries. The findings underscore the importance of interdisciplinary approaches and international collaborations in advancing the understanding of the effects of EE on neural and cognitive outcomes.

From basic behavioral assessments in the late 1960s to complex molecular studies in the 2020s, EE research has significantly evolved, deepening the understanding of how enriched environments impact brain function and behavior. The study has demonstrated that EE research is not only a vibrant and dynamically growing area but also increasingly influential in shaping practices in neuro rehabilitation, animal welfare, and behavioral sciences. Future research is likely to continue exploring these molecular pathways and therapeutic applications, reinforcing the importance of EE in neuroscience and animal welfare studies.

## Data Availability

The original contributions presented in the study are included in the article/Supplementary material, further inquiries can be directed to the corresponding author.
